# Involvement of Transporters in Intestinal Drug–Drug Interactions of Oral Targeted Anticancer Drugs Assessed by Changes in Drug Absorption Time

**DOI:** 10.3390/pharmaceutics14112493

**Published:** 2022-11-17

**Authors:** David Malnoë, Olivier Fardel, Pascal Le Corre

**Affiliations:** 1 Pôle Pharmacie, Service Hospitalo-Universitaire de Pharmacie, CHU de Rennes, 35033 Rennes, France; 2 Laboratoire de Biopharmacie et Pharmacie Clinique, Faculté de Pharmacie, Université de Rennes 1, 35043 Rennes, France; 3 Univ Rennes, CHU Rennes, Inserm, EHESP, Irset (Institut de Recherche en Santé, Environnement et Travail)—UMR_S 1085, 35000 Rennes, France

**Keywords:** intestinal absorption rate, t_max_, mean absorption time, intestinal transporters, mathematical solving, presystemic drug–drug interactions, oral targeted anticancer drugs

## Abstract

(1) Background: Oral targeted anticancer drugs are victims of presystemic pharmacokinetic drug–drug interactions (DDI). Identification of the nature of these DDIs, i.e., enzyme-based or/and transporter-based, is challenging, since most of these drugs are substrates of intestinal and/or hepatic cytochrome P-450 enzymes and of intestinal membrane transporters. (2) Methods: Variations in mean absorption time (MAT) between DDIs and control period (MAT ratios < 0.77 or >1.30) have been proposed to implicate transporters in DDIs at the intestinal level. This methodology has been applied to a large set of oral targeted anticancer drugs (n = 54, involved in 77 DDI studies), from DDI studies available either in the international literature and/or in publicly accessible FDA files. (3) Results: Significant variations in MAT were evidenced in 33 DDI studies, 12 of which could be explained by modulation of an efflux transporter. In 21 DDI studies, modulation of efflux transporters could not explain the MAT variation, suggesting a possible relevant role of influx transporters in the intestinal absorption. (4) Conclusions: This methodology allows one to suggest the involvement of intestinal transporters in DDIs, and should be used in conjunction with in vitro methodologies to help understanding the origin of DDIs.

## 1. Introduction

Since dysfunctions of protein kinases are involved in the pathogenesis of several diseases, including solid or hematologic cancers and cardiovascular, autoimmune, and inflammatory diseases, protein kinase inhibitors (PKI) have triggered a large number of research programs within academia and pharmaceutical companies worldwide, leading to a regular approval of these drugs by the regulatory authorities. A list of PKIs currently in clinical trials is curated in a freely accessible database (http://www.icoa.fr/pkidb, accessed on 29 June 2022) [1]. Nowadays, most of the marketed PKIs are used for the treatment of various solid or hematologic cancers or directed toward inflammatory diseases. These drugs are essentially administered via the oral route, and are victims of presystemic pharmacokinetic drug–drug interactions (DDI), since most of them are substrates of intestinal and/or hepatic cytochrome P-450 (CYP) enzymes (mostly CYP3A4) and membrane transporters [2,3,4,5]. Hence, the characterization of their intestinal bioavailability, and of its factors of variability, is of critical value to optimize drug efficacy, reduce drug toxicity, and improve patient compliance.

Due to the fact that these drugs are not used via the intravenous (IV) route, and to the fact that an IV formulation is rarely available, the characterization of potential intestinal DDIs is not simple, since clearance (CL) measurements are confounded by bioavailability (F) after oral dosing. Recently, Sodhi JK and Benet LZ [6] described a powerful methodology allowing for the discrimination of changes in CL from changes in F in metabolic DDIs. This was made possible by considering that the steady-state volume of distribution (Vss) remains unchanged in metabolic DDIs [7], so changes in apparent Vss (Vss/F) associated to changes in observed apparent clearance (CL/F) may allow one to discriminate changes in F from CL in oral metabolic DDIs. The authors indicate, however, that this method should not be used for drugs when significant systemic transporter-related DDIs are likely, because Vss may be affected by such DDIs. This limitation applies to the PKIs, since most of them are both substrates of CYP450 enzymes and of transporters, leading potentially to complex DDIs.

Complex DDIs may result from different scenarios, including the concurrent inhibition of enzymes and transporters [8], leading to significant challenges for a clear identification of clinical DDIs. Indeed, alteration in the extent of bioavailability may result from metabolic-based DDI by modification in the extent of the fraction of the dose not metabolized in the intestinal (Fg) and/or in the liver (Fh) as well as from transporter-based DDIs by modification of the fraction of the dose entering the enterocyte (Fa) and/or the hepatocyte that may indirectly modify Fg and/or Fh. However, unlike metabolic DDIs, transporter-based DDIs can also result in alterations of the rate of absorption (k_a_) with a decrease in absorption time (decrease in mean absorption time, MAT) linked to the inhibition of intestinal efflux transporters expressed on the apical side, and an increase in absorption time due to the induction of efflux transporter expression (increase in MAT). Based on the theory that significant intestinal transporter interactions should result in an altered rate of absorption of a victim drug, Sodhi and Benet [9] recently proposed a methodology to implicate intestinal transporters in DDI, based on data from clinical studies involving substrates of ATP-binding cassette (ABC) efflux transporters, through inhibition and/or induction of P-glycoprotein (P-gp/*ABCB1*) and/or breast cancer resistance protein (BCRP/*ABCG2*).

The aim of the current study was to apply such methodology to oral targeted anticancer drugs that are narrow therapeutic drugs with potentially complex DDI, to better understand the respective role of metabolism and transporters in their DDI. Data characterizing the absorption time (k_a_ and t_max_) from a panel of 54 drugs were retrieved from DDI studies (n = 101) in published papers and/or from FDA publicly accessible files. Indeed, given that most inhibitors and inducers used in clinical DDI studies are not specific to CYP450 and transporters (or can simultaneously act on both these systems), the conclusions of some of these studies can be challenged.

## 2. Materials and Methods

### 2.1. Data Curation

Given than MAT (or k_a_) was usually not available, t_max_ as well as terminal half-life (t_1/2_) were retrieved when available either from data in tables or after noncompartmental pharmacokinetic analysis from digitization concentration–time profiles. AUC (area under the curve) ratios between DDIs and control periods were calculated from AUC zero-to-infinity for single-dose studies, and from AUC within the dosing interval when studies were performed at steady state. All parameters were reported as ratios from the DDI phase to control phase.

Renal and feces elimination (%, total drug and metabolite(s)), plasma protein binding (%), and blood-to-plasma ratio were also retrieved.

The data allowing for the estimation of the MAT within DDI studies were obtained either from the published literature extracted from Pubmed and/or from freely accessible Food and Drug Administration (FDA) files (section: Clinical Pharmacology and Biopharmaceutics review from multidisciplinary review and from Labeling file) up to 29 June 2021.

### 2.2. Physicochemical and Biopharmaceutical Properties

The main physicochemical properties were estimated from ADMETlab 2.0 (https://admetmesh.scbdd.com/; accessed on 1 September 2021). The parameters are Log D (pH 7.4), Log P, topological polar surface area (TPSA in %), Log S (mol/L), and hydrogen bonding (hydrogen bond donor (HBD) and hydrogen bond acceptor (HBA)). The percentage of polar surface area was calculated from TPSA and from polar surface area (PSA in Å^2^) given by Dragon 6 software (Talete, Milano, Italy) [10].

pH-dependence solubility, solubility at neutral pH, and Biopharmaceutics Classification System (BCS) rating were obtained from FDA-submitted files, and classified according to the United States Pharmacopeia (USP) (i.e., very soluble, freely soluble, soluble, sparingly soluble, slightly soluble, very slightly soluble, and practically insoluble).

Membrane permeability was estimated from ADMETlab 2.0 for (Caco-2 and MDCK permeability).

Determination of the percentage of ionization and of the net charge at neutral (pH 7.40) was performed using MarvinSketch 22.2 (Chemaxon).

The concentration in the intestinal lumen at neutral pH (Igut, mM) was estimated from the maximal solubility at neutral pH (according to USP classification, mg/mL), the usual dose per administration (mg) considering Igut as the maximal soluble dose/250 mL.

### 2.3. Pharmacokinetic Properties

Absolute oral bioavailability (Fabs, %) and BCS data were retrieved from the FDA-submitted files, and when lacking, from the literature [11]. Fabs was available for only 38% of the drugs (28 of 74).

### 2.4. Calculation of Absorption Time

#### 2.4.1. Mathematic Solvation in a Monocompartmental Model with Single Oral Administration

Estimation of the MAT ratio relies on a mathematical solvation using t_1/2_ and t_max_ data. Equation (1) shows the relationship between k_a_, t_max_, and k_e_.

(1) $$t_{\max}=\frac{\ln\left(\frac{k_{a}}{k_{e}}\right)}{k_{a}-k_{e}}$$

**Equation (1** **): Expression of t_max_ according to a monocompartmental model with single oral administration** **.**

From Equation (1), in order to extract k_a_ and express it in terms of k_e_ and t_max_, the Lambert function W defined in Equation (2) must be used.

(2) $$a\times e^{a}=Z\;\to \;a\;=\;W\left(Z\right)$$

**Equation (2** **): Lambert function W definition.**

We set:

$$\begin{array}{c} a\;=-k_{a}\times t_{\max}\\ X\;=k_{e}\times t_{\max} \end{array}$$

where X is strictly positive for t_max_ and k_e_ different from 0.

$$Z=\frac{-X}{e^{X}}$$

The Lambert function has two branches W_0_ and W_−1_. Thus, for all values of Z between 0 and −1/e, W(Z) takes two solutions in the reals. For X < 1, W(Z) takes its solution in the alternative branch W_−1_, and for X ≥ 1, W(Z) takes its solution in the main branch W_0_.

Equation (1) is transformed as follows to match the Lambert function expression and k_a_ is expressed in terms of k_e_ and t_max_ in the resulting Equation (3).

(3) $$\begin{array}{c} e^{\left(-k_{a}\times t_{\max}\right)}\times \left(-k_{a}\times t_{\max}\right)=\frac{-k_{e}\times t_{\max}}{e^{k_{e}\times {\;t}_{\max}}}\\ -k_{a}\times t_{\max}=\;W\left(\frac{-k_{e}\times t_{\max}}{e^{k_{e}\times {\;t}_{\max}}}\right)\\ k_{a}=\frac{W\left(\frac{-k_{e}\times t_{\max}}{e^{k_{e}\times {\;t}_{\max}}}\right)}{-t_{\max}} \end{array}$$

**Equation (3): Expression of k_a_ according to a monocompartmental model with single oral administration using the Lambert function W.**

Since the Lambert function cannot be expressed by the usual functions, it is therefore necessary to resort to approximations by sequence limit (i.e., by asymptotic expansion or by algorithmic approach).

#### 2.4.2. Solvation of the Main Branch W_0_ of Lambert Function

Halley’s method makes it possible to approximate W_0_ (Z) for all Z and for n tending towards infinity. This is a faster and more accurate generalization of Newton’s method (Equation (4)). The sequence quickly converges to W_0_ for n tending towards infinity [12].

(4) $$W_{n+1}=W_{n}-\frac{W_{n\;}\times {\;e}^{W_{n}}-Z}{e^{W_{n}}\left(W_{n}\right)-\frac{\left(W_{n}+2\right)\left(W_{n}\times {\;e}^{W_{n}}-\;Z\right)}{2W_{n}+2}};W_{0}=1$$

**Equation** **(4): Estimation of W_0_ (Z) by Halley’s method that rapidly converges to W_0_ (Z) for n → +** **∞ for all Z.**

#### 2.4.3. Solvation of the Alternative Branch W_−1_ of Lambert Function

It is possible to approximate the W_−1_ branch of the Lambert function with precision by an algorithmic approach, which gives the Equation (5) [13].

(5) $$W_{-1}\left(Z\right)=\ln\left(-Z\right)-2\alpha^{-1}\times \left\{1-{\left[1+\alpha {\left(-\frac{1+\ln\left(-Z\right)}{2}\right)}^{\frac{1}{2}}\right]}^{-1}\right\};\mathrm{with}\alpha =0.3205$$

**Equation (5** **): Estimation of W_−1_ (Z) by an approximation derived from a logarithmic approach.**

#### 2.4.4. Mathematic Solvation in a Monocompartmental Model with Repeated Oral Administration

t_max_ was calculated considering a monocompartmental model after repeated oral administration. Considering τ as the administration interval (h), Equation (6) shows the relationship between k_a_, k_e_, t_max_, and τ.

(6) $$t_{\max}=\frac{\ln\left(\frac{k_{a}\left(1-e^{-k_{e}\times \;\tau }\right)}{k_{e}\left(1-e^{-k_{a}\times \;\tau }\right)}\right)}{k_{a}-k_{e}}$$

**Equation (6): Expression of t_max_ according to a monocompartmental model with repeated oral administration.**

The expression of k_a_ as a function of k_e_, t_max_ and τ from Equation (6) is quite complex, so it requires an approximate solution method by iteration. The algorithm written in Python for this issue is available in the Supplementary Material (Figure S1).

#### 2.4.5. Digitalization of Concentration–Time Profiles

When t_1/2_ or t_max_ were not both available, a digitization of published concentration–time profiles of victim drug was used to estimate the missing data (half-life and/or t_max_) using WebPlotDigitizer Version 4.4^®^ (https://automeris.io/WebPlotDigitizer/; accessed on 20 december 2020) and subsequently analyzed using Pkanalix2020R1^®^ (Lixoft University).

We adopted the following strategy:

1. t_1/2_ and t_max_ published: solving for MAT using multiple-dose equation;
2. t_1/2_ and t_max_ published: solving for MAT using simple-dose equation;
3. Data missing: noncompartmental analysis to retrieve data and solving for MAT using multiple-dose equation;
4. Data missing: noncompartmental analysis to retrieve data and solving for MAT using simple-dose equation;
5. Data missing: MAT cannot be estimated.

#### 2.4.6. Robustness of t_max_ Estimation

MAT ratios displaying changes above 30% (i.e., MAT ratios < 0.77 or >1.30) are considered as indicators of potentially clinically significant intestinal transporter drug–drug interactions [9].

Since the estimation of MAT is highly dependent on the quality of determination of t_max_ between the two periods of the studies (DDI and control arms), we estimated the relevance of this estimation by checking the sampling schedule used in DDI studies. Two sampling points before t_max_ value were considered relevant to estimate that t_max_ determination allowed enough precision, especially for profiles with rapid absorption.

Furthermore, a rapid analysis of the MAT estimation method showed that rather small variations of t_max_ could lead to significant variations in MAT (and MAT ratio), while variation in elimination half-life had a much lower impact on the MAT ratio. Hence, we performed simulations of MAT (and of MAT ratio) by using variation in t_max_ (from ±10%, ±25% and ±50%) reported from the different studies. These variations were applied to both t_max_ of the control period and of the DDI period using the simple-dose equation iterative solvation. We considered our estimation of MAT ratio as robust if variations from −10% to +10% in t_max_ maintained the MAT ratio outside of the range of 0.77 and 1.30.

## 3. Results

Among the 113 oral targeted anticancer drugs recovered from our literature search, we selected those (n = 81) for which sufficient data studies to allow for the estimation of MAT were anticipated, either accessible from FDA files or from the international literature. Within this set of 81 molecules, 74 had an FDA file, and their pharmacologic class (ATC5 and ATC4 classification), initial approval indication and date by the FDA and the sponsor are reported in the Supplementary Material (Table S1). Their main physicochemical and biopharmaceutic properties are presented in Table 1.

Besides the 71 kinase inhibitors within this set of 81 molecules, 10 drugs with similar physicochemical properties are included (i.e., inhibitors of isocitrate dehydrogenase enzyme IDH-I (n = 2: enasidenib, ivosidenib), hedgehog pathway inhibitors HP-I (n = 3: glasdegib, sonidegib, vismodegib), and poly(ADP-ribose) polymerase inhibitors PARP-I (n = 5: niraparib, olaparib, pamiparib, rucaparib, talazoparib)).

From this set of 81 molecules, DDI studies involving either rifampin (RIF), itraconazole (ITRA), or ketoconazole (KETO) with sufficient information to evaluate MAT ratio were found for 54 molecules involved in 101 DDI studies.

### 3.1. Physicochemical and Biopharmaceutical Properties

The solubility characterization was based on the values of the solubility reported at neutral pH and classified according to the USP classification. The distribution of the solubility is illustrated in Supplementary Material (Figure S2). Two-thirds of the compounds (n = 47) have a solubility rated as practically insoluble (PI, i.e., <0.1 mg/mL), and 88% (n = 65) have a pH-dependent solubility. Hence, most of these compounds (92%, n = 68) were rated as BCS class-2, BCS class-4, or BCS class-2/4.

The mean MW (479 ± 83 daltons), TPSA (97.3 ± 22.8 Å), and Log P (3.6 ± 1.2) were close to the mean values calculated from a large database of PKIs either approved or in clinical trials (MW: 463 daltons, TPSA: 96.6 Å and Log P: 3.5 [14]. The mean (± SD) in silico-estimated Caco-2 and MDCK cell permeability was 9.1 ± 7.1 × 10^−6^ and 15.3 ± 12.6 × 10^−6^ cm/s, respectively.

Data reported in Table 1 show that the oral absolute bioavailability was only available for a relatively small number of molecules (i.e., 28/74 molecules). The mean absolute bioavailability was quite large, with a significant variability (mean ± SD: 56 ± 20%).

**Figure 1:**
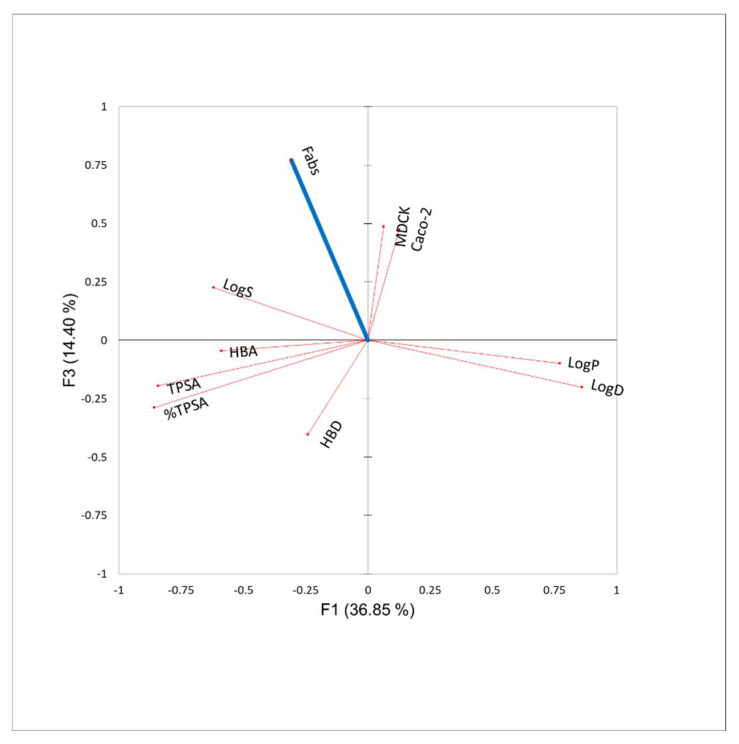
Ordination biplots of principal component analysis (PCA) outputs of the physicochemical space in a series of oral targeted anticancer drugs (n = 28).

PCA analysis showed that the principal components F1 and F3 explained 51.2% of the total data variance in descriptors from the original data set. The variables most correlated to F1 were Log D and Log P (positive correlation), whereas TPSA and %TPSA were negatively correlated to F1 (Figure 1). With regard to the second component (F3), we observed that Fabs was not surprisingly positively correlated with membrane permeability and solubility, and negatively correlated to HBD. However, the absolute values of coefficient of correlations were somewhat small (around 0.5, Table 2). The other variables had a low contribution to Fabs (lower than 6%).

### 3.2. Drug–Drug Interactions

**Figure 2:**
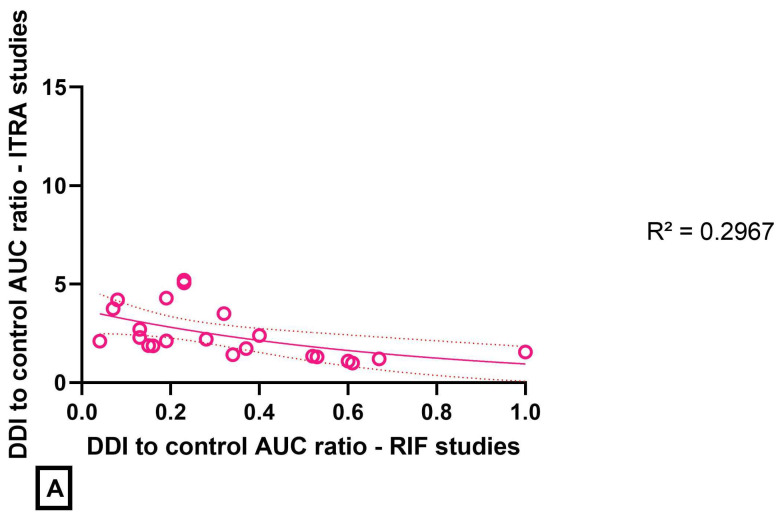

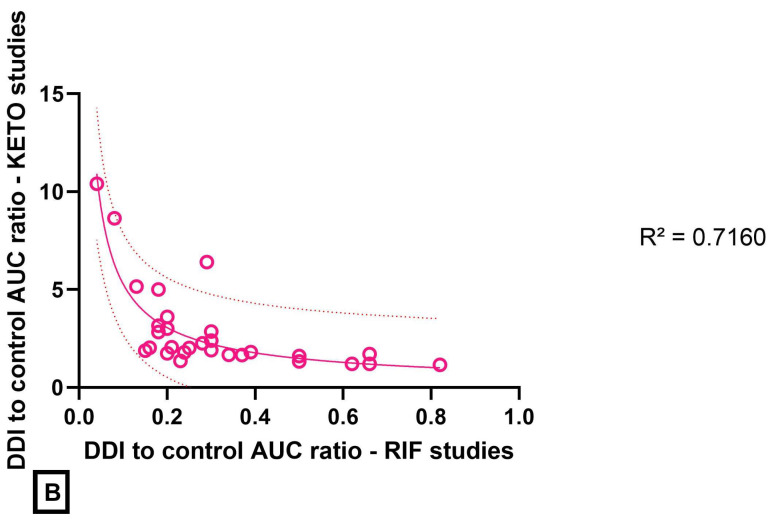
Magnitude of the DDI (estimated by the DDI-to-control AUC ratio) in inhibition DDI studies (with KETO or ITRA) as a function the magnitude of the DDI in induction DDI studies (RIF). (**A**): itraconazole DDI study versus rifampin DDI study. (**B**): ketoconazole DDI study versus rifampin DDI study. The coefficient of determination R^2^ is indicated for each relationship; the 95% confidence interval is represented on each plot.

Based on the DDI-to-control AUC ratio, the intensity of metabolic inhibition (in KETO and ITRA DDI studies) and of metabolic induction (in RIF DDI studies) appeared related. This relationship was more pronounced between KETO and RIF studies (R^2^ = 0.7160, Figure 2B) than between ITRA and RIF studies (R^2^ = 0.2967, Figure 2A).

**Figure 3:**
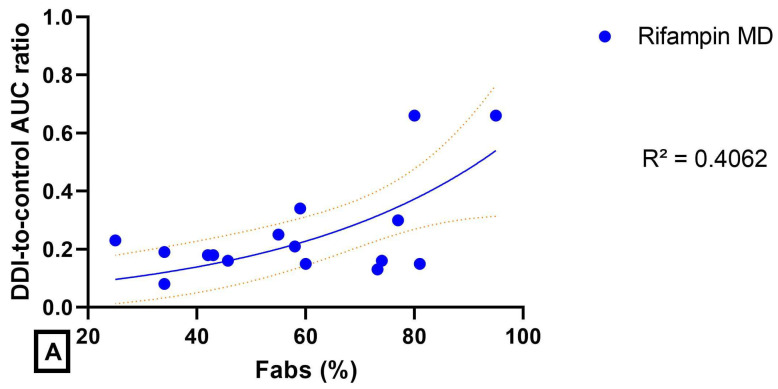

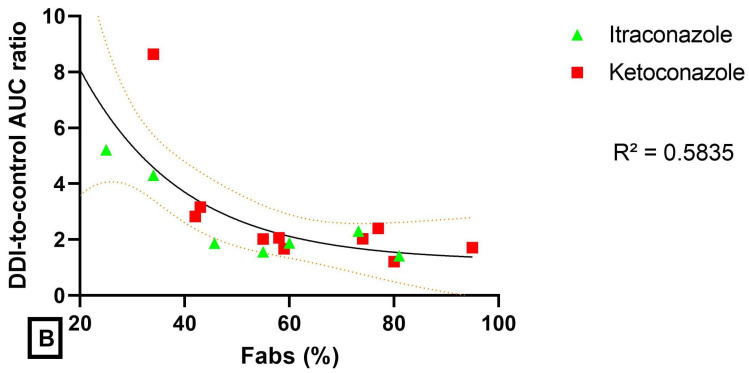
Magnitude of the DDI (estimated by the DDI-to-control AUC ratio) as a function of the oral absolute bioavailability (Fabs, %) (**A**): rifampin DDI studies (blue circle). (**B**): ketoconazole DDI studies (red square) and itraconazole DDI studies (green triangle). The coefficient of determination R^2^ is indicated for each relationship, and the 95% confidence interval is represented on each plot.

The magnitude of the effect of a DDI on the systemic exposure (estimated by the DDI-to-control AUC ratio) with ITRA and KETO decreased with the increase in oral Fabs in a series of drugs including dual substrates of CYP3A4/P-gp (acalabrutinib, axitinib, baricitinib, bosutinib, crizotinib, dabrafenib, duvelisib, erlotinib, gefitinib, glasdegib, larotrectinib, lorlatinib, palbociclib, selpercatinib and tofacitinib) and tazoloparib (P-gp substrate and minimally metabolized). However, the correlation was moderate (R^2^ = 0.5835), as a result of a rather “flat” relationship for drugs with Fabs ranging 40 to 80% (Figure 3B).

The intensity of the DDI with rifampin (as multiple doses) was apparently not influenced by the intensity of Fabs, with DDI-to-control AUC ratio lower than 0.4 for drugs with oral Fabs up to 80% (Figure 3A).

**Figure 4:**
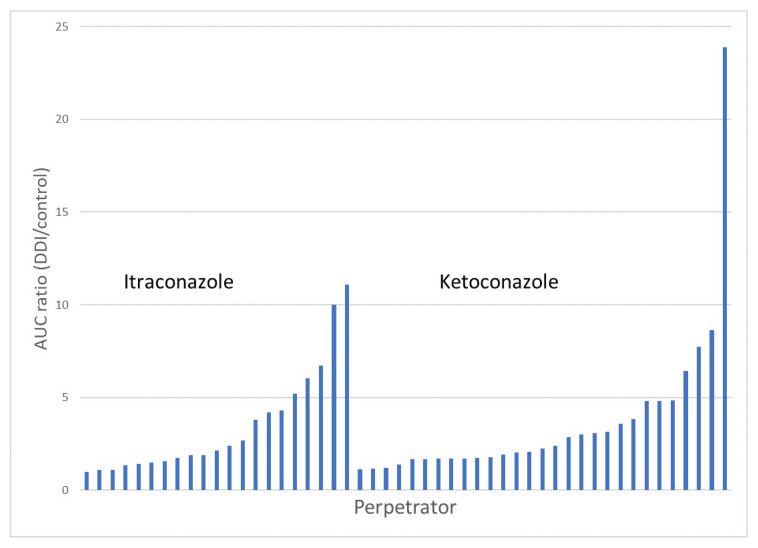
Magnitude of the DDIs (estimated by the DDI-to-control AUC ratio) from KETO and ITRA DDI studies in a series of oral targeted anticancer drugs. The means ± SD of DDI-to-control AUC ratio for KETO and ITRA was 3.73 ± 4.34 (n = 29) and 3.48 ± 2.90 (n = 21).

The magnitude of the effect of a DDI on the absorption rate (estimated by MAT ratio) appeared linked to the intestinal bioavailability (Fabs), both in inhibition and induction DDI studies (Figure 4). In inhibition DDI studies (using ITRA or KETO), the MAT ratio tended to decrease when Fabs decreased for a series of drugs, including dual CYP3A4/P-gp substrates (acalabrutinib, bosutinib, gefitinib, lapatinib, larotrectinib, lorlatinib, palbociclib and pazopanib), and talazoparib (P-gp substrate and minimally metabolized) and alectinib (CYP3A4 substrate and not substrate for P-gp and BCRP). In induction DDI studies (using RIF), the MAT ratio tended to increase when Fabs decreased.

### 3.3. Drug–Drug Interactions

**Figure 5:**
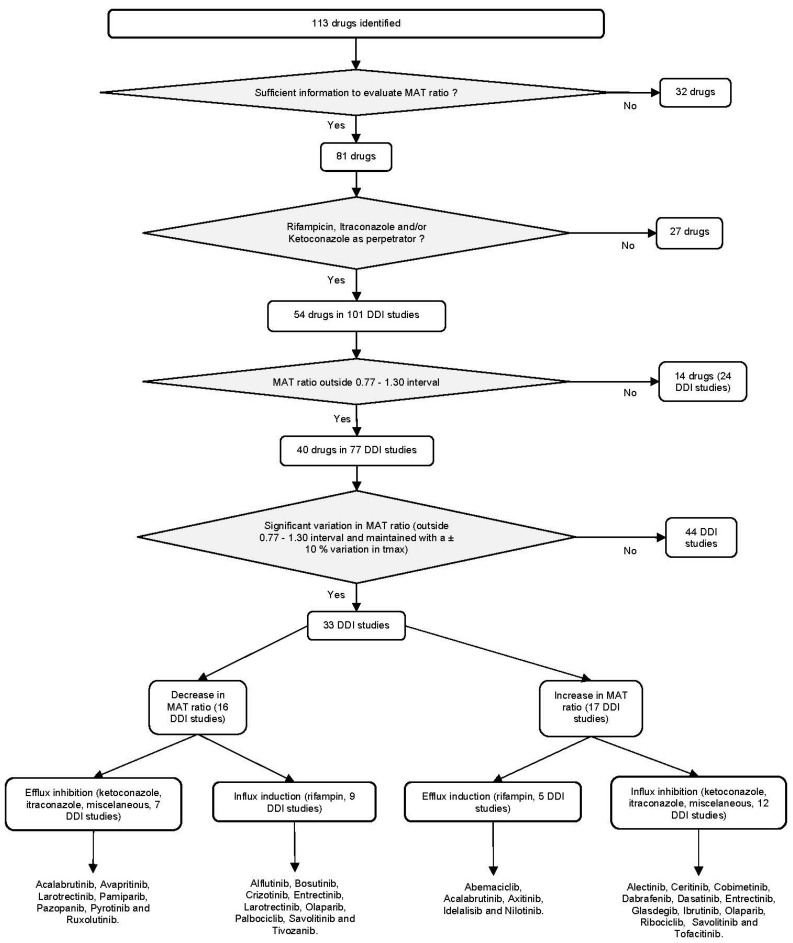
Flow-chart of the studies with potential implication of efflux and influx transporters in DDIs at the intestinal level for oral targeted anticancer drugs.

Finally, we found 54 drugs for which a DDI study with a perpetrator potentially interacting with a transporter (i.e., RIF n = 44, KETO n = 25, ITRA n = 22, and miscellaneous drugs n = 10) was available (see flow-chart in Figure 5).

For 14 of these drugs, involved in 24 DDI studies, there was apparently no modification of the absorption rate (MAT ratio between 0.77 and 1.30), and in most of the DDI studies (n = 20/24), there was no modification in t_max_ (t_max_ ratio = 1, Table 3 bottom).

The top of Table 3 indicates the drugs (n = 40) for which a variation in MAT has been estimated during a DDI study involving either RIF (n = 33), KETO (n = 20), ITRA (n = 15), or with a miscellaneous perpetrator (n = 9). In this series, the BCS classification of the drugs for which a transported-based DDI was suggested (n = 27) was: BCS class-2 (30%), BCS class-4 (26%), BCS class-1 (11%), BCS class-3 (26%), and unknown BCS class (26%).

**Figure 6:**
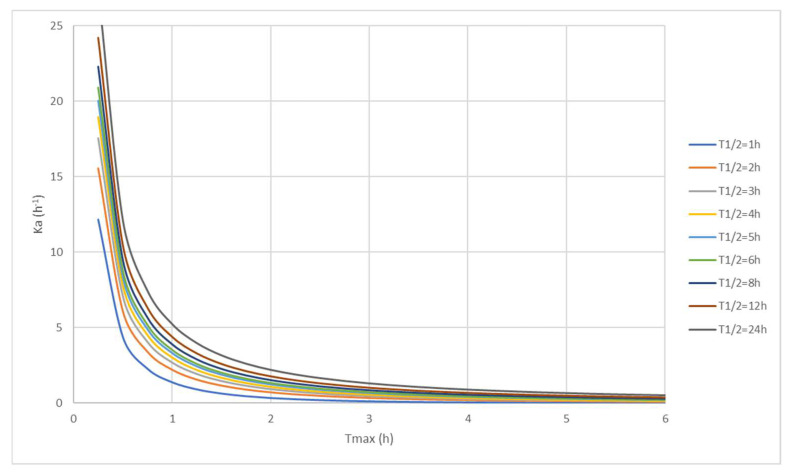
Influence of variations in t_max_ (h) and in t_1/2_ (h^−1^) on the estimation of k_a_. Small variations in t_max_ have greater impact on k_a_ than variations in t_1/2_, especially when t_max_ value are small.

Given that the estimation of MAT is sensible to variations in the determinations of t_max_, rather than variations in t_1/2_ (Figure 6), we decided to add a robustness test by simulating the impact of variations (from ± 10%, ± 25% and ± 50%) in the estimated t_max_ values obtained for both the control and the DDI arms of the studies, even though the MAT ratio was found significant (i.e., >1.30 or <0.77). If the lowest variation (±10% in t_max_) led to a shift in MAT ratio inside the 0.77-to-1.30 range, the robustness of the estimation of the MAT ratio was considered insufficient as a reliant marker of a variation in absorption rate. This can be illustrated by ceritinib in the rifampin DDI study, where the MAT ratio (0.74) shifted into the 0.77-to-1.30 interval. Thus, even if the MAT ratio was <0.77, it was not considered robust enough to be evidence of a variation in the absorption rate. Conversely, considering the rifampin DDI study with palbociclib, the MAT ratio (0.39) remained lower than 0.77, even with variations in t_max_ up to ±50%. The results of the simulations of the variations in t_max_ made for each individual drug are illustrated in Supplementary Material (Figure S3).

**Figure 7:**
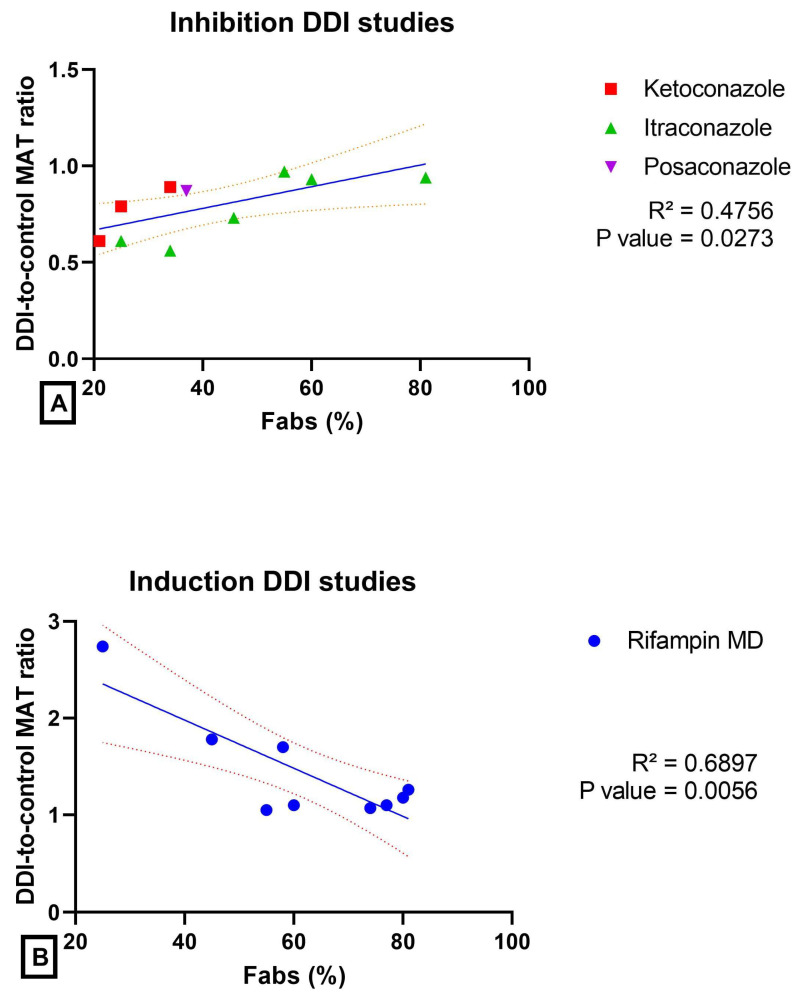
The magnitude of the effect of a DDI on the absorption rate of a drug (estimated by MAT ratio) as a function of the absolute bioavailability (Fabs) in a series of oral targeted anticancer drugs. (**A**): inhibition DDI studies (KETO-ITRA-POSA). (**B**): induction DDI studies (RIF). The coefficient of determination R^2^ and the P value on the linear correlation is indicated for each relationship, and the 95% confidence interval is represented on each plot.

As whole, from 54 DDI studies leading to a MAT ratio >1.30 or <0.77, only 33 of them had a MAT ratio unaffected (i.e., always remaining >1.30 or <0.77) by variations of ±10% in t_max_, and were thus considered as potentially resulting from a variation in the absorption rate. The magnitude of the effect of a DDI on the absorption rate of a drug (estimated by MAT ratio) appeared related to the Fabs of the drugs (Figure 7).

**Table 3:** Involvement of efflux intestinal transporters in DDI studies [15,16,17,18,19,20,21,22,23,24,25,26,27,28,29,30,31,32,33,34,35,36,37,38,39,40,41,42,43,44,45,46,47,48,49,50,51,52,53,54,55,56,57,58,59,60,61,62,63,64,65,66,67,68,69,70,71,72,73,74,75,76,77,78,79,80,81,82,83,84,85,86,87,88,89,90,91,92,93,94,95,96,97,98,99,100,101,102,103,104,105,106,107,108,109].

Drug		Substrate	Inducer	Inhibitor	BCS	F (%)	Population/n	Perpetrator	Victim Drug Dosing (mg)	AUC-DDI/AUC-Control	Control or DDI Period	Tmax	t1/2,z	MAT	MAT Calculation Method	Tmax Ratio	t1/2,z Ratio	MAT Ratio	Absorption Rate (ka)	Potential DDI Mechanism at Enterocyte Level	References
**Top**																					
**Abemaciclib**	*Enzyme*	CYP3A4	-	-	3	45	HS/25	Rifampin	200	0.05	Control	8.0	38.6	2.43	d	1.02	0.33	1.78	**↓**	Efflux induction	[15,16]
	*Transporter*	Pgp and BCRP	-	P-gp, BCRP, OCT2, MATE1 and MATE2-K							DDI	8.2	12.6	4.33							
**Acalabrutinib**	*Enzyme*	CYP3A4	CYP1A2, CYP2B6 and CYP3A4	CYP3A4/5, CYP2C8 and CYP2C9	2	25	HS/16	Itraconazole	50	5.20	Control	0.6	0.7	0.35	d	0.93	1.92	0.61	**↑**	Efflux inhibition	[17,18]
											DDI	0.5	1.4	0.21							
	*Transporter*	P-gp and BCRP	nd	BCRP			HS/24	Rifampin	100	0.23	Control	0.7	1.8	0.30	d	1.05	0.28	2.74	**↓**	Efflux induction	
											DDI	0.8	0.5	0.82							
**Alectinib**	*Enzyme*	CYP3A4	-	nd	4	37	HS/16	Rifampin	600	0.26	Control	6.0	19.2	2.17	b	0.67	0.57	0.71	NS	-	[19,20]
											DDI	4.0	11.0	1.55							
	*Transporter*	-	-	P-gp and BCRP			HS/24	Posaconazole	300	1.75	Control	8.0	18.4	3.39	b	1.00	1.35	0.87	NS	-	
											DDI	8.0	24.8	2.93							
**Alflutinib**	*Enzyme*	CYP3A4	CYP3A4	-	nd	nd	HS/30	Rifampin	80	0.13	Control	4.0	37.1	0.98	b	0.50	0.42	0.53	**↑**	Influx induction	[21]
											DDI	2.0	15.7	0.52							
	*Transporter*	-	nd	nd			HS/30	Itraconazole	400	2.39	Control	3.0	40.6	0.66	b	2.00	1.73	2.08	**↓**	Influx inhibition	[22]
											DDI	6.0	70.3	1.37							
**Avapritinib**	*Enzyme*	CYP3A4 and CYP2C9	CYP3A	CYP34 and CYP2C9	2	nd	HS/nd	Itraconazole	200	4.20	Control	9.7	56.7	2.77	d	0.57	3.24	0.35	**↑**	Efflux inhibition	[23]
	*Transporter*	-	-	P-gp, BCRP, MATE1, MATE2-K, and BSEP							DDI	5.5	183.4	0.98							
**Axitinib**	*Enzyme*	CYP3A4/5, CYP1A2 CYP2C19, and UGT1A1	nd	-	2	58	HS/39	Rifampin	5	0.21	Control	1.5	7.7	0.45	b	1.00	0.32	1.70	**↓**	Efflux induction	[24,25]
											DDI	1.5	2.5	0.76							
	*Transporter*	P-gp, BCRP (weak) and OATP-1B1/1B3	nd	P-gp and BCRP			HS/28	Ketoconazole	5	2.06	Control	1.5	9.4	0.42	b	1.33	1.39	1.32	NS	-	[24,26]
											DDI	2.0	13.1	0.55							
**Bosutinib**	*Enzyme*	CYP3A4	-	-	4	34	HS/22	Rifampin	500	0.08	Control	6.0	33.8	1.73	d	0.50	0.60	0.47	**↑**	Influx induction	[27,28]
											DDI	3.0	20.4	0.81							
	*Transporter*	P-gp	-	P-gp and BCRP			HS/20	Ketoconazole	100	8.64	Control	6.0	46.2	1.56	d	1.00	1.49	0.89	NS	-	[27,29]
											DDI	6.0	69.0	1.38							
**Brigatinib**	*Enzyme*	CYP2C8 and CYP3A4	CYP3A4 and CYP2C’s	-	1	nd	HS/20	Rifampin	90	0.19	Control	2.5	25.1	0.60	b	0.80	0.94	0.76	NS	-	[30,31]
											DDI	2.0	23.7	0.46							
	*Transporter*	P-gp and BCRP	-	P-gp, BCRP, OCT1, MATE1, and MATE2K			HS/20	Itraconazole	90	2.12	Control	2.8	30.5	0.65	b	0.93	1.47	0.82	NS	-	
											DDI	2.6	44.9	0.54							
**Ceritinib**	*Enzyme*	CYP3A4	-	CYP3A4 and CYP2C9	4	nd	HS/19	Rifampin	750	0.30	Control	8.0	38.9	2.44	b	0.75	0.78	0.74	NS	-	[32]
											DDI	6.0	30.3	1.80							
	*Transporter*	P-gp and BCRP	-	OATP1B1-1B3, OAT1 and OCT2			HS/19	Ketoconazole	450	2.86	Control	6.0	47.7	1.54	b	1.67	1.09	1.93	**↓**	Influx inhibition	
											DDI	10.0	52.0	2.97							
**Cobimetinib**	*Enzyme*	CYP3A and UGT2B7	nd	CYP3A and CYP2D6	1	46	HS/15	Itraconazole	10	6.72	Control	2.0	56.8	0.37	d	2.00	2.64	1.89	**↓**	Influx inhibition	[33,34]
	*Transporter*	P-gp	nd	nd							DDI	4.0	150.0	0.69							
**Crizotinib**	*Enzyme*	CYP3A4/5	-	CYP3A	4	43	HS/15	Rifampin	250	0.18	Control	5.0	33.1	1.36	b	0.60	1.46	0.46	**↑**	Influx induction	[35]
											DDI	3.0	48.2	0.63							
	*Transporter*	P-gp	-	P-gp			HS/15	Ketoconazole	150	3.16	Control	5.0	37.1	1.31	b	1.20	1.48	1.12	NS	-	
											DDI	6.0	54.9	1.48							
**Dabrafenib**	*Enzyme*	CYP2C8 and CYP3A4	CYP3A4 and CYP2B6, CYP2C8, CYP2C9, CYP2C19, and UDP glucuronosyltransferases	nd	2	95	P/15	Ketoconazole	75	1.71	Control	1.1	1.9	0.55	c	1.82	1.17	2.53	**↓**	Influx inhibition	[36,37]
	*Transporter*	P-gp and BCRP	nd	OATP1B1, OATP1B3, OAT1/3 and BCRP							DDI	2.0	2.3	1.39							
**Dacomitinib**	*Enzyme*	CYP2D6 and CYP2C9, CYP3A4	-	CYP2D6 and UGT1A1	2	80	H/14	Paroxetine	45	1.37	Control	10.0	90.1	2.48	b	0.80	1.07	0.74	NS	-	[38,39]
	*Transporter*	P-gp and BCRP	nd	P-gp, BCRP and OCT1							DDI	8.0	96.2	1.82							
**Dasatinib**	*Enzyme*	CYP3A4	nd	nd	2	nd	P/18	Ketoconazole	20	4.84	Control	0.4	3.3	0.09	c	4.03	2.64	4.93	**↓**	Influx inhibition	[40,41]
											DDI	1.5	8.7	0.44							
	*Transporter*	P-gp and BCRP	nd	P-gp and BCRP OATP1B1/1B3			HS/20	Rifampin	100	0.18	Control	1.0	4.7	0.31	b	1.00	0.72	1.14	NS	-	
											DDI	1.0	3.4	0.35							
**Entrectinib**	*Enzyme*	CYP3A4	-	CYP3A4	2	nd	P/10	Rifampin	600	0.23	Control	5.0	15.5	1.81	d	0.15	0.29	0.11	**↑**	Influx induction	[42,43]
											DDI	0.7	4.5	0.20							
	*Transporter*	P-gp (weak)	-	P-gp, BCRP, OATP1B1, and MATE1			P/10	Itraconazole	100	6.04	Control	1.6	20.1	0.36	b	2.90	2.76	2.94	**↓**	Influx inhibition	[42]
											DDI	4.6	55.5	1.06							
**Fedratinib**	*Enzyme*	CYP3A4, CYP2C19	nd	CYP3A4, CYP2D6 and CYP2C19	2	nd	HS/7	Ketoconazole	300	3.06	Control	3.0	77.0	0.56	b	0.83	1.23	0.77	NS	-	[44,45]
											DDI	2.5	95.0	0.43							
	*Transporter*	P-gp	nd	P-gp, BCRP, OATP1B1, OATP1B3, OCT2, MATE 1 and MATE-2K			HS/7	Ketoconazole	50	3.85	Control	1.5	112.0	0.23	b	1.00	1.17	0.97	NS	-	
											DDI	1.5	131.0	0.22							
**Fuzuloparib**	*Enzyme*	CYP3A4	CYP1A2, CYP2B6 and CYP3A4	nd	nd	nd	HS/16	Rifampin	50	0.11	Control	3.0	10.8	1.03	b	0.67	0.19	1.35	NS	-	[46]
	*Transporter*	nd	nd	nd							DDI	2.0	2.1	1.39							
**Glasdegib**	*Enzyme*	CYP3A4, CYP2C8 and UGT1A9	-	-	4	77	HS/14	Ketoconazole	200	2.40	Control	1.0	18.3	0.20	b	2.00	1.09	2.35	**↓**	Influx inhibition	[47]
											DDI	2.0	20.0	0.48							
	*Transporter*	P-gp and BCRP	-	P-gp, BCRP, MATE1 and MATE-2K			HS/12	Rifampin	100	0.30	Control	1.5	13.4	0.37	b	0.83	0.38	1.10	NS	-	[47,48]
											DDI	1.3	5.1	0.41							
**Ibrutinib**	*Enzyme*	CYP3A4 and CYP2D6	nd	nd	nd	nd	HS/11	Itraconazole	140	10.00	Control	2.0	4.7	0.84	b	1.50	0.81	2.17	**↓**	Influx inhibition	[49]
											DDI	3.0	3.8	1.81							
	*Transporter*	nd	nd	P-gp (GIT)			HS/18	Ketoconazole	120	23.90	Control	1.4	2.6	0.69	d	1.34	1.75	1.16	NS	-	[50]
											DDI	1.9	4.6	0.80							
**Idelalisib**	*Enzyme*	Aldehyde oxidase, CYP3A4 and UGT1A4	nd	nd	2	nd	HS/12	Rifampin	150	0.24	Control	1.8	5.8	0.62	b	0.86	0.31	1.53	**↓**	Efflux induction	[51]
	*Transporter*	P-gp and BCRP	nd	P-gp - OATP1B1-1B3							DDI	1.5	1.8	0.96							
**Lapatinib**	*Enzyme*	CYP3A4, CYP3A5, CYP2C19 and CYP2C8	nd	CYP3A4	4	25	HS/20	Ketoconazole	100	3.57	Control	4.0	9.6	1.66	b	1.00	1.68	0.79	NS	-	[52,53]
											DDI	4.0	16.0	1.32							
	*Transporter*	P-gp and BCRP	nd	P-gp and BCRP			HS/23	Carbamazepine	250	0.28	Control	4.0	10.2	1.61	b	0.75	0.98	0.66	NS	-	[53]
											DDI	3.0	10.0	1.07							
**Larotrectinib**	*Enzyme*	CYP3A4	-	-	1	34	HS/12	Rifampin	100	0.19	Control	1.0	2.9	0.39	d	0.46	0.41	0.49	**↑**	Influx induction	[54,55]
											DDI	0.5	1.2	0.19							
							HS/12	Rifampin - SD	100	0.19	Control	1.0	2.9	0.39	d	0.99	0.66	1.22	NS	-	[55]
	*Transporter*	P-gp and BCRP and OATP1A2 (weak)	-	-							DDI	1.0	1.9	0.48							
							HS/12	Itraconazole	100	4.30	Control	0.9	2.5	0.37	d	0.89	3.11	0.56	**↑**	Efflux inhibition	
											DDI	0.8	7.7	0.20							
**Lenvatinib**	*Enzyme*	CYP3A and aldehyde oxidase	CYP3A	CYP2C8, CYP1A2, CYP2B6, CYP2C9, CYP2C19, CYP2D6, CYP3A and UGT1A1	nd	nd	HS/14	Rifampin	24	0.82	Control	2.0	22.0	0.47	b	1.25	0.82	1.42	NS	-	[56,57]
											DDI	2.5	18.2	0.68							
							HS/15	Rifampin - SD	24	1.36	Control	2.0	22.0	0.48	b	1.00	0.98	0.99	NS	-	
	*Transporter*	P-gp and BCRP	nd	nd							DDI	2.0	21.5	0.48							
							HS/18	Ketoconazole	5	1.16	Control	3.0	29.0	0.73	b	1.00	1.01	1.00	NS	-	[57]
											DDI	3.0	29.2	0.72							
**Motesanib**	*Enzyme*	nd	nd	nd	nd	nd	P/12	Ketoconazole	50	1.66	Control	0.9	5.7	0.24	a	0.85	1.38	0.73	NS	-	[58]
	*Transporter*	nd	nd	nd							DDI	0.8	7.8	0.18							
**Nilotinib**	*Enzyme*	CYP3A4	nd	CYP3A4/5, CYP2C8, CYP2C9, CYP2D6, and UGT1A1	4	nd	HS/15	Rifampin	400	0.20	Control	3.0	18.6	0.84	b	1.33	0.78	1.63	**↓**	Efflux induction	[59,60]
											DDI	4.0	14.6	1.37							
	*Transporter*	P-gp and BCRP	nd	P-gp and BCRP			HS/25	Ketoconazole	200	3.01	Control	4.0	15.2	1.34	b	1.00	2.15	0.76	NS	-	
											DDI	4.0	32.7	1.02							
**Olaparib**	*Enzyme*	CYP3A	CYP3A - CYP2B6	CYP3A - UGT1A1	4	nd	P/18	Rifampin	300	0.13	Control	1.5	13.2	0.37	b	0.53	1.20	0.42	**↑**	Influx induction	[61,62]
											DDI	0.8	15.8	0.16							
	*Transporter*	P-gp	-	P-gp, BCRP, OATP1B1, OCT1, OCT2, OAT3, MATE1, and MATE2K			P/53	Itraconazole	100	2.70	Control	1.0	15.0	0.22	b	1.46	1.04	1.60	**↓**	Influx inhibition	
											DDI	1.5	15.6	0.36							
**Palbociclib**	*Enzyme*	CYP3A4 - SULT2A1	-	CYP3A (tile- dep)	2	45.7	HS/12	Itraconazole	125	1.87	Control	8.1	22.1	3.14	b	0.91	1.54	0.73	NS	-	[63,64]
											DDI	7.4	33.9	2.28							
	*Transporter*	P-gp and BCRP	-	P-gp, BCRP and OCT1 - OAT1, OAT3, OCT2, OATP1B1/3 (low)			HS/15	Rifampin	125	0.16	Control	8.0	22.6	3.06	b	0.38	0.34	0.39	**↑**	Influx induction	
											DDI	3.0	7.8	1.20							
**Pamiparib**	*Enzyme*	CYP3A and 2C8	nd	nd	nd	nd	P/12	Itraconazole	20	0.99	Control	2.0	9.3	0.62	b	0.50	1.20	0.37	**↑**	Efflux inhibition	[65]
											DDI	1.0	11.2	0.23							
	*Transporter*	-	nd	nd			P/11	Rifampin	60	0.57	Control	2.0	13.4	0.543	b	1.00	0.57	1.23	NS	-	
											DDI	2.0	7.7	0.67							
**Pazopanib**	*Enzyme*	CYP3A4,CYP1A2 and CYP2C8	CYP3A4 and CYP2B6	CYP1A2, CYP2A6, CYP2B6, CYP2C8, CYP2C9, CYP2C19, CYP2D6, CYP2E1, CYP3A4, UGT1A1	nd	21	P/16	Ketoconazole	400	1.66	Control	4.0	41.5	0.94	c	0.87	3.91	0.61	**↑**	Efflux inhibition	[66,67]
	*Transporter*	P-gp and BCRP	nd	OATP1B1							DDI	3.5	162.3	0.58							
**Pexidartinib**	*Enzyme*	CYP3A4 and UGT1A4	CYP2B6	CYP2B6	2	nd	HS/16	Rifampin	600	0.37	Control	2.5	24.2	0.61	b	0.80	1.19	1.43	NS	-	[68]
											DDI	3.0	16.8	0.87							
	*Transporter*	-	-	MATE1, MATE2-K, OATP1B1, OATP1B3 and OATP2B1			HS/16	Itraconazole	600	1.73	Control	2.5	24.2	0.61	b	0.80	1.19	0.72	NS	-	
											DDI	2.0	28.8	0.43							
**Pyrotinib**	*Enzyme*	CYP3A4	nd	nd	nd	nd	HS/18	Itraconazole	80	11.10	Control	5.0	12.9	1.98	b	1.00	4.43	0.58	**↑**	Efflux inhibition	[69]
	*Transporter*	nd	nd	nd							DDI	5.0	57.3	1.15							
**Ribociclib**	*Enzyme*	CYP3A4 and FMO3 (minor)	-	CYP3A, CYP1A2 and CYP2E1	4	nd	HS/24	Ritonavir	400	3.21	Control	1.5	30.6	0.29	d	4.20	1.79	5.28	**↓**	Influx inhibition	[70,71]
											DDI	6.1	54.8	1.51							
	*Transporter*	P-gp and BCRP	nd	BCRP, OCT2, MATE1, and human BSEP - P-gp, OATP1B1/B3, OCT1, and MATEK2 (low)			HS/24	Rifampin	600	0.11	Control	2.1	31.5	0.44	d	0.86	0.39	1.08	NS	-	
											DDI	1.8	12.4	0.47							
**Ruxolitinib**	*Enzyme*	CYP3A4 and CYP2C9	-	-	1	nd	HS/12	Rifampin	50	0.30	Control	1.0	3.2	0.36	b	1.00	0.50	1.44	NS	-	[72,73]
											DDI	1.0	1.6	0.52							
							HS/14	Erythromycin	10	1.27	Control	1.5	4.1	0.58	b	0.67	1.10	0.54	**↑**	Efflux inhibition	
	*Transporter*	-	-	-							DDI	1.0	4.5	0.31							
							HS/16	Ketoconazole	10	1.91	Control	1.0	3.5	0.35	b	1.00	1.60	0.83	NS	-	
											DDI	1.0	5.6	0.29							
**Savolitinib**	*Enzyme*	**CYP3A4**-CYP1A2-UGT1A4-UGT2B15-aldehyde oxydase	nd	nd	nd	nd	HS/18	Rifampin	600	0.39	Control	4.0	7.1	1.95	b	0.75	0.99	0.65	**↑**	Influx induction	[74]
											DDI	3.0	7.0	1.26							
	*Transporter*	P-gp	nd	nd			HS/15	Itraconazole	200	1.08	Control	2.5	4.2	1.26	b	1.60	1.10	2.06	**↓**	Influx inhibition	
											DDI	4.0	4.6	2.59							
**Selumetinib**	*Enzyme*	CYP3A4, CYP2C19, CYP1A2, CYP2C9, CYP2E1, CYP3A5 and UGT1A1/3	-	-	4	62	HS /24	Itraconazole	25	1.49	Control	1.0	8.2	0.25	b	1.00	1.71	0.86	NS	-	[75]
											DDI	1.0	14.0	0.22							
							HS/22	Rifampin	75	0.49	Control	1.3	9.3	0.34	b	0.79	0.72	0.81	NS	-	
	*Transporter*	P-gp and BCRP	-	OAT3							DDI	1.0	6.7	0.27							
							HS/22	Fluconazole	25	1.50	Control	1.0	8.2	0.25	b	1.50	1.20	1.62	NS	-	
											DDI	1.5	9.8	0.41							
**Sunitinib**	*Enzyme*	CYP3A4	-	-	4	nd	HS caucasian/14	Rifampin	50	0.21	Control	8.5	48.5	2.44	b	0.92	0.33	1.44	NS	-	[76]
											DDI	7.8	15.9	3.52							
							HS asian/12	Rifampin	50	0.21	Control	7.9	49.5	2.20	b	0.91	0.29	1.49	NS	-	
											DDI	7.2	14.5	3.27							
	*Transporter*	P-gp and BCRP	-	P-gp and BCRP			HS caucasian/14	Ketoconazole	10	1.70	Control	7.9	41.2	2.34	b	0.91	1.06	0.86	NS	-	
											DDI	7.2	43.5	2.03							
							HS asian/14	Ketoconazole	10	1.70	Control	8.9	43.2	2.71	b	0.97	1.02	0.95	NS	-	
											DDI	8.6	43.9	2.57							
**Tivozanib**	*Enzyme*	CYP3A4 and CYP1A1	-	-	nd	nd	HS/25	Ketoconazole	1.5	1.12	Control	10.0	117.0	2.29	b	0.75	0.96	0.71	NS	-	[77,78]
											DDI	7.5	112.0	1.62							
	*Transporter*	-	nd	BCRP			HS/27	Rifampin	1.5	0.47	Control	10.0	121.0	2.27	b	0.30	0.45	0.27	**↑**	Influx induction	
											DDI	3.0	54.0	0.61							
**Tofacitinib**	*Enzyme*	**CYP3A4**, CYP2C19	-	-	3	74	HS/12	Ketoconazole	10	2.03	Control	0.5	2.9	0.14	b	2.00	1.34	2.33	**↓**	Influx inhibition	[79,80]
											DDI	1.0	3.9	0.33							
							HS/12	Rifampin	30	0.16	Control	0.5	3.1	0.15	d	0.94	0.67	1.07	NS	-	[79]
	*Transporter*	P-gp	nd	Pgp, OCT2 and OATP1B1 (low)							DDI	0.5	2.1	0.16							
							HS/22	Ciclosporin A	10	1.73	Control	0.5	3.2	0.13	d	1.04	1.20	1.00	NS	-	
											DDI	0.5	3.8	0.13							
**Upadacitinib**	*Enzyme*	CYP3A4 and CYP2D6	-	-	1	nd	HS/12	Rifampin - SD	12	1.07	Control	2.9	6.5	1.18	b	0.97	0.91	1.05	NS	-	[81,82]
											DDI	2.8	5.9	1.24							
							HS/12	Rifampin	12	0.39	Control	2.9	6.5	1.18	b	0.91	0.75	1.30	NS	-	
	*Transporter*	P-gp and BCRP	nd	P-gp, BCRP and OATP1B1 (weak)							DDI	2.8	4.9	1.54							
							HS/11	Ketoconazole	3	1.75	Control	1.1	8.5	0.29	b	0.82	0.87	0.80	NS	-	
											DDI	0.9	7.4	0.23							
**Vemurafenib**	*Enzyme*	CYP3A4	-	CYP1A2, 2A6, 2C9, 2C19, 2D6, and 3A4/5	4	nd	P/23	Rifampin	960	0.53	Control	4.0	30.0	1.05	b	1.00	0.40	1.42	NS	-	[83,84]
	*Transporter*	P-gp and BCRP	-	P-gp and BCRP							DDI	4.0	12.0	1.49							
**Bottom**																					
**Afatinib**	*Enzyme*	minimal	-	-	1 or 3	nd	HS/22	Ritonavir	20	1.48	Control	4.0	35.9	0.99	b	1.00	0.95	1.02	NS	-	[85,86]
											DDI	4.0	34.1	1.01							
	*Transporter*	P-gp and BCRP	nd	P-gp and BCRP			HS/22	Rifampin	40	0.66	Control	6.0	32.8	1.75	b	1.00	1.10	0.97	NS	-	
											DDI	6.0	36.0	1.69							
**Baricitinib**	*Enzyme*	CYP3A4	nd	nd	3	80	HS/18	Rifampin	10	0.66	Control	1.0	7.7	0.26	b	1.00	0.62	1.18	NS	-	[87]
											DDI	1.0	4.8	0.31							
	*Transporter*	P-gp, BCRP, OAT3 and MATE2-K	nd	OAT-2			HS/34	Ketoconazole	10	1.21	Control	1.0	6.6	0.27	b	1.00	1.10	0.97	NS	-	
											DDI	1.0	7.3	0.26							
**Cabozantinib**	*Enzyme*	CYP3A4	CYP1A1	CYP3A4, CYP2C8 and CYP2C9	nd	nd	HS/25	Rifampin	140	0.23	Control	4.0	111.0	0.74	b	0.75	0.25	1.00	NS	-	[88,89]
											DDI	3.0	27.7	0.74							
	*Transporter*	-	nd	P-gp			HS/27	Ketoconazole	140	1.38	Control	4.0	122.0	0.72	b	1.00	1.18	0.97	NS	-	
											DDI	4.0	144.0	0.70							
**Erdafitinib**	*Enzyme*	CYP2C9 and CYP 3A4	CYP3A4 (TD)	CYP3A4 (TD)	1	nd	HS/17	Itraconazole	4	1.34	Control	2.0	59.1	0.36	b	1.00	1.31	0.94	NS	-	[90,91]
	*Transporter*	P-gp	nd	P-gp and OCT--2							DDI	2.0	77.5	0.34							
**Gefitinib**	*Enzyme*	CYP3A4	nd	CYP2C19,CYP2D6, CYP2C9, CYP3A4, CYP1A2 and CYP2C8	3	60	HS/24	Itraconazole	250	1.88	Control	5.0	30.7	1.40	b	1.00	1.25	0.93	NS	-	[92]
											DDI	5.0	38.5	1.30							
	*Transporter*	P-gp and BCRP	nd	P-gp and BCRP			HS/18	Rifampin	500	0.15	Control	3.0	33.8	0.70	b	1.00	0.61	1.16	NS	-	
											DDI	3.0	20.7	0.81							
**Ivosidenib**	*Enzyme*	CYP3A4	CYP2B6, CYP2C8, CYP2C9, and CYP3A4	nd	2	nd	P/21	Itraconazole	250	2.69	Control	4.0	60.7	0.85	b	1.00	2.31	0.83	NS	-	[93,94]
	*Transporter*	P-gp	nd	P-gp and OAT3							DDI	4.0	140.2	0.70							
**Lorlatinib**	*Enzyme*	CYP3A4 and UGT1A4	CYP3A4 and CYP2B6	CYP3A4	1	81	HS/12	Rifampin	100	0.15	Control	1.5	21.2	0.33	b	1.00	0.48	1.26	NS	-	[95]
											DDI	1.5	10.2	0.41							
	*Transporter*	P-gp	nd	nd			HS/12	Itraconazole	100	1.42	Control	1.5	23.1	0.32	b	1.00	1.29	0.94	NS	-	
											DDI	1.5	29.8	0.30							
**Neratinib**	*Enzyme*	CYP3A4 and FMO	-	CYP3A4 and CYP2B6	2	nd	HS/22	Ketoconazole	240	4.81	Control	6.0	11.7	2.78	b	1.00	1.55	0.80	NS	-	[96,97]
	*Transporter*	nd	nd	P-gp, BCRP and OCT1							DDI	6.0	18.0	2.23							
**Peficitinib**	*Enzyme*	nd	nd	nd			HS/24	Verapamil	150	1.27	Control	2.0	9.5	0.61	b	1.00	1.46	0.87	NS	-	[98]
	*Transporter*	P-gp	nd	OCT--1 and MATE1							DDI	2.0	13.9	0.54							
**Ponatinib**	*Enzyme*	CYP3A4, CYP2C8 and CYP2D6	-	-	2	nd	HS/19	Rifampin	15	0.38	Control	6.0	27.0	1.88	b	1.00	0.73	1.14	NS	-	[99,100]
											DDI	6.0	19.6	2.15							
	*Transporter*	P-gp and BCRP (weak)	nd	P-gp, BCRP and BSEP			HS/19	Ketoconazole	15	1.78	Control	6.0	35.3	1.70	b	1.00	1.06	0.98	NS	-	[99,101]
											DDI	6.0	37.4	1.67							
**Sonidegib**	*Enzyme*	CYP3A4	-	CYP2B6 and CYP2C9	2	nd	HS/16	Rifampin	800	0.28	Control	2.0	124.0	0.31	b	1.00	0.67	1.08	NS	-	[102,103]
											DDI	2.0	82.9	0.34							
	*Transporter*	-	nd	BCRP			HS/15	Ketoconazole	800	2.25	Control	2.0	124.0	0.31	b	1.00	3.38	0.82	NS	-	
											DDI	2.0	419.0	0.26							
**Talazoparib**	*Enzyme*	minimal	-	-	2 or 4	55	P /19	Itraconazole	0.5	1.56	Control	1.0	101.0	0.14	b	1.00	0.17	0.97	NS	-	[104,105]
											DDI	1.0	118.0	0.14							
	*Transporter*	P-gp and BCRP	-	-			P/17	Rifampin	1	1.00	Control	1.0	92.1	0.15	b	1.00	0.88	1.02	NS	-	
											DDI	1.0	80.6	0.15							
**Vandetanib**	*Enzyme*	CYP3A4 and FMO1/3	CYP1A2, CYP2C9 and CYP3A4	CYP2D6 and CYP2C8	2	nd	HS /18	Itraconazole	300	1.09	Control	5.0	209.2	0.85	b	1.00	1.13	0.98	NS	-	[106,107]
											DDI	5.0	235.5	0.83							
	*Transporter*	P-gp	nd	P-gp and OCT2			HS/12	Rifampin	300	0.60	Control	6.0	217.6	1.05	b	0.83	0.53	0.92	NS	-	
											DDI	5.0	116.3	0.96							
**Zanubrutinib**	*Enzyme*	CYP3A4	nd	CYP2C8, CYP2C9, and CYP2C19	2 or 4	nd	HS/20	Rifampin	320	0.07	Control	2.0	6.8	0.71	b	1.00	0.80	1.18	NS	-	[108,109]
											DDI	2.0	4.8	0.83							
	*Transporter*	P-gp	nd	OCT--2			HS/19	Itraconazole	320	3.78	Control	1.5	2.2	0.82	b	1.33	1.95	1.07	NS	-	
											DDI	2.0	4.3	0.88							

Ratios of oral pharmacokinetic DDI parameters (reported as interaction/control) and substrate specificities, and the inhibition or inducing potential of victim drugs for metabolic enzymes and xenobiotic transporters of 54 drugs, for which a DDI study with a perpetrator potentially interacting with a transporter (i.e., RIF n = 44, KETO n = 25 and ITRA n = 22, and miscellaneous drugs n = 10) were available. Pharmacokinetic values reported in the table are based on published average values. **Top** indicates the drugs (n = 40, 77 DDI studies) for which a significant variation in MAT has been evidenced during a DDI study involving either RIF (n = 33), KETO (n = 20), ITRA (n = 15) or with a miscellaneous perpetrator (n = 9). **Bottom** indicates the drugs (n = 14, 24 DDI studies) for which there was apparently no modification of the absorption rate (MAT ratio between 0.77 and 1.30. MAT values in bold are those considered as a relevant marker of a variation in the absorption rate: MAT ratio outside the 0.77–1.30 interval and remaining outside this interval following simulation variations in t_max_ (±10%). The methodology of calculation is described in detail in the Materials and Methods section. The increase in MAT ratio is shown in green and the decrease in MAT ratio in blue. The darker color is for the potential influx implication and the lighter color represents the potential efflux implication. MAT calculation method: (a) Data published: solving for MAT using multiple-dose equation; (b) Data published: solving for MAT using simple-dose equation; (c) Data missing: noncompartmental analysis to retrieve data and solving for MAT using multiple-dose equation; (d) Data missing: noncompartmental analysis to retrieve data and solving for MAT using simple-dose equation.

## 4. Discussion

As shown in Table 1, the oral absolute bioavailability was only available for few drugs of our sample set (i.e., 28 on 74 molecules). The mean absolute bioavailability was quite large, with a significant variability (mean ± SD: 56 ± 20%), and 15 of 28 drugs had an oral bioavailability above 50%. The application of the Lipinski “rule of 5” [110] indicated that 57% of the compounds (n = 40) were likely to have favorable absorption or permeation properties, bearing in mind that drugs that are substrates of transporters could be exceptions to the rule if intestinal transporters significantly influence intestinal absorption.

PCA analysis showed that the variables most contributing to Fabs were not surprisingly apparent: membrane permeability (positively correlated) and HBD (negatively correlated). However, the absolute values of correlation coefficients were somewhat small (ranging from 0.4 to 0.5, Table 2). This may result from the fact that the fraction of the drug metabolized was not integrated as a variable since it was not available.

### 4.1. Drug–Drug Interactions

Besides their potential to interact with CYP3A4, KETO, ITRA, and RIF also interact with transporters at the intestinal level, at least as strong inhibitors of P-gp (KETO and ITRA), and as strong inducer (RIF in multiple dosing) of P-gp. This potential double interaction at the CYP3A4 and P-gp level makes it difficult to evaluate the contribution of these mechanisms to the interaction (particularly the contribution of P-gp) in DDI studies for drugs that are substrates of both these biological systems. This was the case for half of the drugs in our sample set (40/81 drugs were substrates of both CYP3A4 and of an efflux transporter, Table 3). Furthermore, the intensity of the DDI resulting from CYP3A4 modulation should overcome the impact of P-gp modulation (or of another efflux transporter). Indeed, considering talinolol (P-gp substrate and not metabolized CYP3A4), the effect of rifampin was not so high, with a DDI-to-control AUC ratio of 0.66 [111].

When the contribution of CYP3A4 to the overall elimination of a drug is substantial, regulatory agencies have initially recommended the use of KETO and of RIF (as multiple doses), as inhibitor and inducer, respectively, in in vivo DDI studies. While KETO is a strong, selective, and reversible inhibitor of CYP3A4, concerns related to its liver toxicity have made it no longer usable in clinical trials, and several replacement inhibitors have been proposed (i.e., ITRA, ritonavir, and clarithromycin) [112]. ITRA and clarithromycin have been recommended by the FDA, and ITRA, KETO, ritonavir, and clarithromycin are currently proposed by the European Medicines Agency (EMA) [113,114]. Based on its high intensity of CYP3A4 inhibition (estimated by the fold increase in midazolam AUC), ITRA has emerged and is now widely used as a CYP3A4 reversible inhibitor, both in the gut wall and the liver, with recommendations for its clinical use [112,115]. However, there are still some debates on the use of ITRA, which is not considered as strong as KETO regarding inhibition of CYP3A, and reinstating KETO as an index inhibitor for CYP3A has been proposed [116]. The argument in favor of a stronger inhibition with KETO can be challenged in the light of our data obtained with a large panel of drugs. Indeed, the comparison of the DDI-to-control AUC ratio from KETO and ITRA DDI studies indicates that the intensity of the interaction is quite similar (Figure 4). Indeed, the mean DDI-to-control AUC ratio for KETO and ITRA was 3.73 ± 4.34 (n = 29) and 3.48 ± 2.90 (n = 21), respectively.

Based on the DDI-to-control AUC ratio, the magnitude of DDI within KETO-RIF DDI and ITRA-RIF DDI studies was related. The relationship appeared more straightforward between KETO and RIF (Figure 2B) than between ITRA and RIF (Figure 2A). The coefficients of determination indicate that the magnitude of inhibition is not so indicative of the magnitude of induction and vice versa, and this is more especially clear for ITRA. These elements question the relevance of the extrapolation of DDI to clinical situations where different inhibitors or inducers may be used in patients. This strengthens the use of modeling strategies in application files to regulatory authorities, mainly static and dynamic physiologically based pharmacokinetic (PBPK) modeling approaches, even though there are still gaps in their prediction accuracy [117]. However, confidence of regulatory agencies in PKPB model prediction of induction is not particularly high, especially when a drug has multiple pathways and/or undergoes competing DDI mechanisms.

The magnitude of the DDI with ITRA and KETO logically decreased with the increase in oral Fabs. However, the correlation was moderate (R^2^ = 0.5835) as a result of a rather “flat” relationship for drugs with Fabs ranging 40 to 80% (Figure 3B). The magnitude of the DDI with RIF was apparently not influenced by the intensity of Fabs with a DDI-to-control AUC ratio lower than 0.4 for drugs with oral absolute bioavailability up to 80% (Figure 3A). However, it should be noticed that a better correlation, and a lower scattering, would have been obtained by using the extent of the fraction of the drug metabolized by CYP3A4/5 (fm, CYP3A4/5) instead of Fabs that depends on both fm and of the fraction absorbed.

The magnitude of the effect of a DDI on the absorption rate of a drug (estimated by MAT ratio) appeared related to its absolute bioavailability (Figure 7). In inhibition DDI studies, the MAT ratio decreased with the decrease in Fabs. This is not unlikely, given that low Fabs can result from either a low permeability and/or from a low solubility. Indeed, the impact of the inhibition of an efflux transporter is much more significant for drugs with low intestinal permeabilities. It should be noticed that drugs with low permeability (belonging to the BCS class 3 and 4) were overrepresented in this series of drugs with variations in MAT ratio and documented Fabs. For drugs with low solubility, the impact of the inhibition of an efflux transporter is more apparent, given that the saturation of the transporter is unlikely.

In induction DDI studies, there was a trend in the MAT ratio to increase when Fabs decreased, suggesting that the lower the bioavailability, the higher the impact of an efflux transporter inhibition on the absorption rate. Similarly, inhibition DDI studies have clearly shown that the systemic exposure and the absorption rate increased with the decrease in bioavailability (Figure 3B and Figure 7B).

The complexity of the interplay between CYP3A4 and P-gp makes it difficult to estimate the contribution of the modulation of efflux transporter to the variations in the systemic exposure. This interplay between CYP3A4 and P-gp at the intestinal level creates a functional synergy that tends to restrict the systemic exposure of oral drugs that are dual substrates. This results from reabsorption cycling, which increases the chance of a drug to be metabolized by CYP3A4, and secondly by a decrease in the intracellular concentration of drugs in the enterocytes, avoiding CYP3A4 saturation. Pharmacokinetic modeling may be used to estimate the contribution of P-gp to systemic exposure of CYP3A4 metabolized drugs. Based on induction studies with RIF in a series of drugs, including kinase inhibitors, it has been estimated that the contribution of P-gp to the decrease in AUC was 1.2-fold to 1.6-fold for CYP3A4/P-gp dual substrates in comparison to only considering CYP3A4 induction [118].

### 4.2. Transporters and Variations in Absorption Rate

Transporter-based DDIs can result in the modification of the rate of absorption (k_a_) of drug substrates of intestinal transporters expressed on the apical side of enterocytes, leading to an increase or to a decrease in MAT depending on the nature of the transporter (efflux or influx) and of the interaction (inhibition or induction).

Within the set of 54 drugs, we found that there was apparently no modification of the absorption rate (MAT ratio between 0.77 and 1.30) for 14 drugs, with no modification in t_max_ (t_max_ ratio = 1, Table 3 bottom). This was not unlikely for cabozantinib, neratinib, and sonidegib, that were not known to be substrate of P-gp and/or BCRP, as well as for ponatinib considered as a weak P-gp/BCRP substrate (Table 3). Despite being substrates of P-gp and/of BCRP, erdafitinib and lorlatinib are BCS class 1 drugs, so their absorption is unlikely to be influenced by efflux transporters (either by bypass or by saturation of the efflux transporters by intestinal concentrations of these drugs). The absorption rate of afatinib (BCS class 1/3), of baricitinib and of gefitinib (BCS class 3), which are both substrates of P-gp and/or BCRP, may not be influenced, as a potential saturation of the efflux transporters may occur. However, the MAT of ivosidenib and vandetanib (BCS class 2), and of talazoparib and zanubrutinib (BCS class 2/4), which are P-gp or BCRP substrates, should theoretically be sensitive to efflux transporter effects, given their low solubility. This was not the case in DDI studies with ITRA or KETO. Given that these drugs are practically insoluble and the usual recommended dose of their maximum concentration in gut lumen at neutral pH is lower that 0.21 mM (especially for tazaloparib, Supplementary Material Table S2), a saturation of efflux transporter is unlikely for these drugs. Hence, the lack of apparent effects might result from opposite effects towards efflux and uptake transporters on drug absorption.

For 40 drugs within the set (74%), a significant variation in MAT ratio was evidenced in either one or in all inhibition/induction DDI studies. For axitinib, a significant variation was evidenced in the MAT ratio in the RIF DDI study (MAT ratio = 1.70) but not in the KETO study (MAT ratio = 1.32). Considering alflutinib, a significant variation in MAT ratio was evidenced in both the RIF and KETO studies (0.53 and 2.08, respectively). As a whole, for these 40 drugs, a significant variation in MAT ratio (i.e., >1.30 or <0.77) was evidenced within 70% of the DDI studies (i.e., in 54 of all the 77 DDI studies).

Given that estimations of MAT are sensible to variations in the determinations of t_max_, as shown in Figure 6 and to a lesser extent to variations in t_1/2_, we simulated the impact of variations in t_max_ on the estimation of MAT. This allowed us, as a more conservative approach, to exclude DDIs where the MAT ratio (although outside the 0.77-to-1.30 interval) was estimated to be not reliant enough as an indicator of variation in the absorption rate (i.e., involving a DDI at the transporter level). These exclusions were observed for several drugs for which the MAT ratio was close to the limits of the 0.77–1.30 interval (Supplementary Material Figure S3).

Within the DDI studies where the MAT ratio was considered significant (n = 33, resulting either from an increase or a decrease in absorption rate), we found that the induction or the inhibition of an efflux transporter (P-gp or BCRP) may explain such variations in 12 DDI studies (Figure 5). Indeed, the decrease in the absorption rate of abemaciclib (P-gp substrate) by RIF was consistent with an induction of P-gp (MAT ratio of 1.78). The induction of intestinal efflux transporters (by RIF on multiple dosing) should increase drug cycling between the enterocytes and gut lumen, thus leading to an increase in absorption time (i.e., increase in MAT). Conversely, the decrease in MAT ratio (0.61) in the KETO DDI study of acalabrutinib (P-gp and BCRP substrate) is consistent with an inhibition of intestinal efflux transporters increasing the absorption rate.

However, in 21 of the 33 DDI studies (Figure 5), potential variations in the absorption rate could not be related to a modulation in efflux transporters. For nine studies involving RIF, the decrease in MAT (suggesting an increase in absorption rate) was related either with an efflux inhibition or with an influx induction. Since RIF is not known to be an efflux inhibitor in vivo (in multiple doses), we therefore hypothesized the involvement of an influx transporter that would be induced by RIF. For 12 studies involving KETO and ITRA, an increase in MAT (suggesting a decrease in absorption rate) was observed. Such prolongation in absorption is compatible with either an efflux induction or with an influx inhibition. As KETO and ITRA are well established efflux inhibitors (and not efflux inducers) [119,120]), we therefore hypothesized that this could result from an influx inhibition. Indeed, the increase in the MAT of cobimetinib (P-gp substrate) by ITRA (MAT = 1.89, and 2-fold increase in t_max_) indicated a decrease in absorption rate that could result from the inhibition of an influx transporter. These observations were also made with the antiplatelet agent ticagrelor and RIF (MAT and t1/2 ratios reduced by 50%), suggesting the induction of solute carriers (SLCs) such as organic anion-transporting polypeptide transporters (OATPs/*SLCOs*) or a competitive inhibition of P-gp by RIF when given at the same time with the victim drug [121].

Besides OATPs, organic ion transporters belonging to the SLC22 family, i.e., organic anion transporters (OATs), organic cation transporters (OCTs), and organic cation/carnitine transporters (OCTNs), play a major role in human physiology and in pharmacokinetics through the absorption and disposition of drugs [122]. The role of influx transporters in the uptake of oral targeted anticancer drugs is of increasing significance, and translation to humans should be made with caution since there are some discrepancies in observations from cellular models, questioning the most relevant transfected cells to be used. As a prototypic drug, imatinib uptake was successively shown to be mainly driven by OCT1 (*SLC22A1*), then by OCT2 (*SLC22A2*), and finally by OATP1A2 (*SLCO1A2*) [123,124]. In an overview, for 15 tyrosine kinase inhibitors (TKIs) as substrates and/or inhibitors of influx transporters, OATP1B1/1B3 (*SLCO1B1/1B3*) were most cited as influx transporters [123]. Furthermore, transport within enterocytes should not only be considered from the apical to basolateral side, but basolateral to apical movement may also play a crucial role in the absorption. Hence, the involvement of transporters at the basolateral interface should also be considered. Moreover, the exact location of some SLC transporters at the apical or basolateral pole of enterocytes remains debated, notably for OATP2B1 (*SLCO2B1*) [125].

Within influx intestinal transporters, transporters of organic cations should be considered given the chemical structure of our drugs on interest (i.e., bearing positive charge at pH = 7.40 for most of them). Various SLCs handle organic cations with different molecular structures: OCT1, OCT2, and OCT3 (*SLC22A3*) (These SLCs are facilitated transporters that are independent of sodium or proton gradients, i.e., exclusive facilitative diffusion transporters), while OCTN1 (*SLC22A4*) and OCTN2 (*SLC22A5*) are efficient transporters of zwiterrions [126]. These transporters are involved in the absorption and/or excretion of organic cations at the intestinal, liver, and renal levels. Polyspecific OCTs (OCT1, OCT2, OCTN1, OCTN2) and ENT4 (*SLC29A4*), are involved in the enterocyte uptake of cationic drugs, and they display some overlap in substrate selectivity [127]. OCT1, OCT3, OCTN1, and OCTN2 are thought to be located at the apical membrane of the enterocytes, and are predominantly involved in the first step of organic cation absorption.

It should be noticed that there is little information on their relative abundance at the apical or basolateral membrane of enterocytes, and on their functional relevance for the uptake of individual drugs. mRNA abundance measurement showed an expression of OCTN1 and OCTN2, and a low but relevant expression of OCT1, OCT3 and the additional cation transporter multidrug and toxin extrusion protein (MATE) 2-K (*SLC47A2*), while no expression of mRNA was detected for OCT2 and MATE1 (*SLC47A1*) [126]. Intestinal abundance of OCT1 has been estimated as rather low and close to OATP2B1 expression, corresponding to 1–2% of all transporters in intestinal segments in humans [128]. Moreover, there are no data on the second step of the intestinal absorption of cationic drugs, allowing the uptake or secretion at the basolateral membrane, and cation transporters at the basolateral side of the enterocytes have not yet been identified [127].

However, the role of transporters of organic cations in the uptake of kinase inhibitors should be considered. Indeed, competitive inhibitions using intestinal Caco-2 cell experiments showed that OCT3 was involved in the uptake of gefitinib and OCT1, OCT2, and OCT3 were involved in sunitinib and crizotinib uptake, while erlotinib uptake was not modified [129]. Moreover, various TKIs have been shown to interact with OCT3 [130]. However, the analysis of drug transport with cell lines may be complicated with the intracellular accumulation evidenced in Caco-2 cells [123] that may result from lysosomal accumulation for cationic drugs [131]. Indeed, given their chemical structure of cationic hydrophobic drugs, such compounds can diffuse in the lysosome by passive diffusion and potentially via an additional mechanism using P-gp located on the lysosomal membrane [132]. Variability in intracellular distribution was shown among a set of seven PKIs, with a high uptake for sunitinib and crizotinib [129].

The role of SLCs as influx transporters is also dependent on the net charge and polarity of the drug, which may influence the relative contribution of passive diffusion and influx transport. Indeed, the intestinal absorption of sunitinib—a strong cationic drug at pH 7.40—may be more dependent on influx transport rather than passive diffusion, as opposed to nilotinib, whose net charge at pH 7.40 is close to zero (net charge of +0.98 and +0.03 for sunitinib and nilotinib, respectively; Table 1).

When extrapolating in vitro experiments to in vivo situations for the role of organic cation transporters at the intestinal level, it should be kept in mind that immunolocalization and pharmacokinetics have suggested OCT1 expression in the basolateral membrane [133], while other results have supported an apical localization in intestinal epithelia cells [134]. Hence, further investigations are necessary to clarify the positioning of the different transporters of organic cations in the enterocytes, notably because their inhibition at the apical and at the basolateral poles should have opposite effects on the rate of drug absorption.

Within DDI studies, interaction of KETO, ITRA, and RIF with SLC uptake transporters should be considered. KETO has been shown to inhibit the standard OCT substrate ASP+ uptake by 82.3% in an OCT1 inhibition assay using HEK-OCT1 cells [135]. Furthermore, KETO has been identified as a potent inhibitor of OCT1 with an IC_50_ value of 2.6 µM [136]. Moreover, KETO also inhibits OATP1B1, OATP1B3, OAT1 (*SLC22A6*), OAT3 (*SLC22A8*), OCT1, OCT2, and MATE1, with IC_50_ values lower than 1 µM [119]. While ITRA only inhibited OCT1 (IC_50_=0.74 µM), its metabolites (hydroxy-itraconazole and keto-itraconazole) had low IC_50_ values for OATP1B1, OATP1B3, OCT1, and MATE1.

Another triazole antifungal agent (isavuconazole) blocks OCT1, OCT2, and MATE1 (or a combination thereof), with a rather mild inhibition intensity in vivo (1.5-fold AUC increase), using metformin as substrate [137]. The influence of isavuconazole on the absorption rate was evidenced with a decrease in MAT ratio (0.71). However, variations of ±10% in k_a_ led to a shift in MAT ratio in the 0.77–1.30 range, so it should not be considered relevant. It should be noticed that such an increase in absorption rate may be consistent with an inhibition of an OCT transporter at the basolateral level in the enterocyte.

RIF may interfere with OCT1, through mediating its upregulation at the intestinal level, as suggested in a DDI study with metformin as the victim drug [138]. The authors indicated that the DDI between RIF and metformin was not consistent with an increase in OCT1 hepatic uptake nor with an OCT2 increase in renal tubular secretion, but rather with a modification in metformin intestinal absorption kinetics, because early metformin plasma concentrations were higher after RIF treatment. However, this assumption should be ruled out because the MAT ratio that we estimated from their data was 1.0, without modification in t_max_ (t_max_ ratio = 0.98). Besides a potential upregulation of OCT1 expression, RIF has been shown to inhibit uptake of the reference OCT1 substrate ASP+ with a low intensity, from 15% [139] to 26.2% [135]. Hence, the role of RIF at the intestinal level may be quite complex, since upregulation of OCT1 expression and inhibition of its activity at the apical level should have opposite effects.

Within our sample set (Table 3), the decrease in MAT reported in KETO/ITRA DDI studies (n = 12) may be consistent a mechanism of uptake inhibition at the abovementioned apical level. The theoretical concentrations of these inhibitors in the GI tract within DDI studies lead to a value in the mM range (around 4 mM and 3 mM for KETO and ITRA) that is much higher than the reported IC_50_ values. However, the contribution of influx transporters in the absorption of oral targeted anticancer drugs should be studied using relevant cellular models to validate our assumptions.

### 4.3. Limitations

The MAT methodology did not allow for an estimation of the contribution of intestinal transporter-based DDIs to the variation in drug exposure, since the vast majority of small oral molecules in cancer studies are substrates of enzymes (mostly CYP3A4) without intravenous pharmacokinetic data. Hence, the clinical relevance of intestinal transporter-based DDIs is yet to be substantiated. Improving knowledge in transporter-based DDIs at the intestinal level should contribute to a complete characterization of transporter-based DDIs at the time of initial NDA application, for which additional efforts from sponsors are expected [140]. This would be of value to health care professionals to foster safe and effective coadministration of these small oral molecules with other co-medications.

The MAT methodology may also have some limitations for drugs with a pronounced distribution process that could be apparent after single oral dosing (i.e., distribution nose), so the equations used may lead to an error in estimation of k_a_. For such drugs, the estimation of k_a_ from steady-state dosing should be preferred, given that at steady state there is much less distribution, and drugs behave as one-compartment model drugs.

Problems of drug solubility at the intestinal level may constitute a bias in the application of the MAT methodology. The drugs of interest were weak basic drugs that typically dissolve at low pH and potentially precipitate at elevated pH (above the pKa), since a majority of them had a pH-dependent solubility. Hence, if the unionized form of the base form is poorly soluble, the absorption may be solubility-dependent, with a zero-order absorption rate not compatible with the assumptions that were made. Such a phenomenon may result in a decrease in the absorption rate, impacting the estimation of MAT. However, if the solubility was a limiting factor for absorption, this phenomenon may similarly impact the absorption rate in both the control and DDI arm of the studies, so variations in MAT should not appear. If we cannot rule out that solubility could be a limiting factor for absorption of such drugs, it seems apparent that this was not the case for most of the drugs, for which a variation in MAT, as well as in t_max_, was reported in DDI studies. Furthermore, inspection of the plasma concentration–time curves did not lead to unexpected shapes, which could be related to a solubility-limited absorption.

From a biopharmaceutic point of view, the relevance of an interaction with the organic cation uptake transporter at the apical level (by induction or inhibition) for BCS Class 2 drugs may be questioned as a result of their high permeability [141], and these potential DDIs should be more relevant for BCS class 3/4 drugs [142].

SLCs handling organic cationic drugs are mainly expressed in clearance organs (The ranking based on relative gene expression in human liver: OCT1 >> OCT3 > OCT2-OCTN1, and in human kidney: OCT2-OCTN2 > OCT1-OCT3 > OCTN1 [143]), so that inhibition at these elimination organs may increase the apparent elimination half-life, which may impact MAT estimation. However, we previously showed that MAT determination was much less sensible to variations in t_1/2_ than in t_max_ values, so a DDI at a systemic level is unlikely to be a confounding factor.

## 5. Conclusions

The MAT methodology introduced by Sodhi and Benet [9] was used to explore the involvement of transporters in DDIs at the intestinal level in a large series of small oral targeted anticancer drugs. In order to avoid an overinterpretation in variations in MAT ratio, we proposed to add a sensitivity test by simulating the influence of variations of t_max_ on the estimation of MAT ratio, to increase the robustness of the MAT ratio estimation.

A subset of DDIs was consistent with induction or inhibition of efflux transporters at the apical level (namely P-gp and/or BCRP) with well-known perpetrators (ITRA, KETO, and RIF). However, a majority of the DDIs were more consistent with a perpetrator effect on influx transporters of cationic drugs at the apical level either by inhibition of the influx by KETO or ITRA, or by an upregulation by rifampin. However, to confirm these assumptions, investigations are necessary to clarify the apical and/or basolateral positioning of different SLCs in the enterocytes, because the inhibition at the apical and at the basolateral level should have opposite effects on the rate of drug absorption. These investigations are particularly required for small oral molecules in cancers, given the complexity of their intestinal absorption, resulting in a potential interplay between the pH-dependent solubility, the intrinsic permeability, and the relative contribution of passive diffusion and of efflux/influx transporter-mediated passage that may be influenced by the percentage of ionization.

Moreover, this MAT methodology is useful to confirm the involvement of transporters in DDIs at the intestinal level, and should be used in conjunction with in vitro methodologies to help understand the origin of DDIs at the intestinal level and their clinical relevance. This may help sponsors for a complete evaluation of transporter-based DDIs at the time of initial NDA approval.

## Figures and Tables

**Table 1:** Physicochemical and biopharmaceutic properties of 74 oral anticancer drugs. BCS (Biopharmaceutical Classification System); MW (molecular weight); nd (no data available); nHA (number of hydrogen bond acceptors); nHD (number of hydrogen bond donors); PI (practically insoluble); PSA (polar surface area); S (soluble); TPSA (topological polar surface area); VSS (very slightly soluble).

Drug	MW (g.mol^−1^)	LogS	USP Solubility at Neutral pH	Solubility pH-Dependent	Estimated Concentration in the Intestine at Neutral pH (mM)	Ionisation at Neutral pH (%)	Net Charge at pH 7.40	Caco-2 Permeability (10^−6^ cm/sec)	MDCK Permeability (10^−6^ cm/sec)	Fabs (%)	BCS	LogD	LogP	nHA	nHD	TPSA (Å²)	TSA (Å²)	%TPSA
Abemaciclib	507	−2.2	PI	yes	0.20	77.9	0.78	12.1	14.0	45	3	3.0	3.6	8	1	78	630	12.4
Acalabrutinib	466	−3.7	PI	yes	0.21	0.2	0.00	2.0	4.8	25	2	2.2	2.0	9	3	122	564	19.4
Afatinib	486	−4.5	S	yes	0.33	96.2	0.98	19.9	17.6	nd	1 or 3	3.1	3.2	8	2	92	585	14.6
Alectinib	483	−6.4	PI	yes	0.21	60.9	0.61	1.0	12.7	37	4	4.0	5.5	6	1	72	626	11.5
Alpelisib	442	−4.4	PI	yes	0.23	28.8	-0.29	13.1	13.5	nd	2	2.4	3.2	7	3	104	527	16.6
Avapritinib	499	−4.2	PI	yes	0.20	93.0	0.93	15.3	20.3	nd	2	2.5	3.6	10	2	106	590	16.9
Axitinib	387	−4.3	PI	yes	0.05	0.2	0.00	9.1	15.8	58	2	3.6	3.8	5	2	71	466	11.2
Baricitinib	371	−3.2	PI	yes	0.02	0.4	0.04	1.7	5.6	80	3	1.1	0.4	9	1	121	428	19.1
Binimetinib	441	−5.8	PI	yes	0.23	0.6	0.00	26.4	17.3	nd	nd	2.3	3.7	7	3	88	452	14.0
Bosutinib	530	−5.4	PI	yes	0.19	81.0	0.81	2.3	12.2	34	4	3.5	4.0	8	1	86	662	13.7
Brigatinib	584	−3.5	VS	no	0.82	93.2	0.93	12.2	11.0	nd	1	2.9	3.0	9	2	92	742	14.6
Cabozantinib	502	−6.6	PI	yes	0.20	3.0	0.03	5.5	15.4	nd	nd	3.4	4.3	8	2	99	818	15.7
Capmatinib	412	−4.6	nd	yes	nd	0.1	0.00	18.1	36.8	nd	2	2.7	3.1	7	1	85	463	13.5
Ceritinib	558	−4.3	PI	yes	0.18	99.8	1.00	3.2	14.9	nd	4	4.5	4.3	8	3	112	722	17.7
Cobimetinib	532	−4.7	VSS	yes	0.45	99.6	1.00	18.0	59.8	46	1	3.6	4.7	5	3	65	524	10.2
Crizotinib	450	−3.9	PI	yes	0.22	99.8	1.21	1.7	5.9	43	4	3.6	3.8	6	3	79	521	12.5
Dabrafenib	520	−4.4	PI	yes	0.19	63.7	-0.64	1.5	87.2	95	2	1.9	4.2	7	3	112	572	17.7
Dacomitinib	470	−4.3	PI	yes	0.21	93.4	0.96	5.2	24.4	80	2	3.5	4.3	7	2	83	570	13.1
Dasatinib	489	−4.3	PI	yes	0.20	38.4	0.39	13.5	12.5	nd	2	2.9	2.8	9	3	110	600	17.4
Duvelisib	417	−4.0	PI	yes	0.24	0.3	0.01	9.5	8.0	42	nd	2.9	2.7	7	2	92	468	14.6
Enasidenib	473	−3.6	PI	yes	0.21	0.0	0.00	30.9	24.4	57	nd	3.1	3.3	8	3	115	526	18.3
Encorafenib	540	−4.7	PI	yes	0.19	8.2	-0.08	1.6	13.1	nd	2	1.2	3.1	11	3	143	656	22.7
Entrectinib	561	−5.7	PI	yes	0.18	70.9	0.71	2.5	7.9	nd	2	3.8	4.9	8	3	89	671	14.1
Erdafitinib	447	−4.0	SS	yes	0.07	99.5	1.00	10.8	8.2	nd	1	3.4	4.4	8	1	77	599	12.3
Erlotinib	393	−4.7	VSS	yes	1.53	0.5	0.03	21.5	15.8	59	2	3.0	2.5	7	1	78	525	12.4
Fedratinib	525	−4.3	PI	yes	0.19	97.7	0.99	7.3	15.8	nd	nd	3.5	4.1	9	3	115	695	18.2
Fostamatinib	581	−3.0	SS	yes	0.69	100.0	-1.94	6.5	9.5	nd	nd	0.9	0.9	15	3	190	674	30.1
Gefitinib	447	−4.5	PI	yes	0.22	22.1	0.25	10.8	25.8	60	3	3.5	3.8	7	1	72	536	11.4
Gilteritinib	553	−2.4	SPS	yes	0.87	92.2	0.92	2.6	3.6	nd	nd	2.6	2.5	11	4	124	747	19.7
Glasdegib	374	−4.1	SS	yes	2.14	15.6	0.15	1.4	4.7	77	4	2.7	2.4	7	3	97	477	15.4
Ibrutinib	441	−3.4	PI	yes	0.23	0.6	0.01	3.9	9.3	nd	nd	3.4	3.2	8	2	100	539	15.8
Idelalisib	415	−3.6	PI	yes	0.24	0.2	0.00	9.5	7.7	nd	2	2.2	1.6	8	2	105	469	16.6
Imatinib	494	−3.3	VS	yes	1.62	73.5	0.74	2.7	7.3	nd	nd	3.1	3.8	8	2	90	636	14.2
Infigratinib	560	−4.6	PI	yes	0.18	87.5	0.87	18.6	19.4	nd	2	3.4	4.0	10	2	98	695	15.6
Ivosidenib	583	−4.4	PI	yes	0.17	0.0	0.00	10.0	31.5	nd	2	2.5	3.1	9	1	119	656	18.9
Lapatinib	581	−4.1	PI	nd	0.17	42.2	0.43	2.0	12.5	nd	4	3.5	4.3	8	2	110	662	17.4
Larotrectinib	428	−5.0	FS	yes	0.93	0.0	0.00	12.1	18.2	34	nd	2.8	3.3	8	2	86	494	13.6
Lenvatinib	427	−7.1	VSS	yes	0.22	1.0	0.01	5.2	7.6	nd	nd	2.9	3.1	8	4	116	494	18.3
Lorlatinib	406	−3.3	VSS	yes	0.98	2.0	0.17	2.6	7.0	81	nd	1.8	1.3	8	2	111	484	17.6
Midostaurin	571	−7.4	PI	nd	0.18	0.0	0.00	9.4	41.3	nd	2	4.0	5.5	8	1	78	632	12.3
Neratinib	557	−4.6	PI	yes	0.18	96.2	0.96	7.9	9.4	nd	nd	3.3	3.9	9	2	116	712	18.3
Nilotinib	530	−4.0	PI	yes	0.19	3.2	0.03	7.7	11.2	nd	4	3.9	4.9	8	2	101	599	16.0
Niraparib	320	−3.1	SS	no	3.75	99.8	1.00	3.2	5.0	73	2	2.3	2.3	5	3	73	398	11.6
Olaparib	435	−4.8	VSS	no	2.30	0.3	0.00	6.4	12.3	nd	4	2.2	2.2	7	1	86	511	13.7
Osimertinib	500	−4.7	VSS	yes	0.64	96.7	0.97	3.8	9.1	nd	nd	3.5	3.8	9	2	91	668	14.4
Palbociclib	448	−3.3	VSS	yes	1.12	96.7	0.97	2.6	2.4	46	2	2.5	2.2	9	2	108	563	17.2
Pazopanib	438	−4.2	PI	yes	0.23	0.2	0.01	3.2	3.0	21	nd	3.1	3.3	9	3	122	522	19.4
Pemigatinib	488	−3.9	PI	yes	0.11	3.1	0.03	7.8	20.9	nd	2	2.8	3.0	9	1	83	564	13.2
Pexidartinib	418	−4.3	PI	yes	0.24	18.7	0.19	8.8	18.5	nd	2	3.9	4.4	5	2	70	441	11.1
Ponatinib	533	−5.3	PI	yes	0.19	62.7	0.63	4.4	15.2	nd	2	4.2	5.3	7	1	66	626	10.4
Pralsetinib	533	−3.3	PI	yes	0.19	0.2	0.00	13.9	5.1	nd	2	3.3	4.0	11	3	139	675	22.0
Regorafenib	483	−6.8	PI	no	0.21	0.1	0.00	3.9	16.0	nd	2	3.6	5.3	7	3	92	517	14.7
Ribociclib	435	−1.4	VSS	yes	2.30	96.7	0.97	3.0	1.9	nd	4	2.6	2.2	9	2	94	550	15.0
Ripretinib	510	−7.4	PI	yes	0.20	2.9	0.22	4.2	13.0	nd	2 or 4	3.9	5.2	7	3	91	552	14.5
Rucaparib	323	−4.1	SS	no	7.42	98.8	0.99	3.9	9.1	36	nd	2.6	2.8	4	3	57	376	9.0
Ruxolitinib	306	−3.6	VSS	yes	0.26	0.4	0.04	19.4	5.1	nd	1	3.0	2.6	6	1	83	385	13.2
Selpercatinib	526	−3.0	VSS	yes	1.22	7.1	0.07	20.4	27.7	73	4	2.9	3.5	10	1	112	676	17.8
Selumetinib	458	−5.9	SS	yes	0.22	0.6	0.00	28.7	21.5	62	4	2.6	4.2	7	3	88	471	14.0
Sonidegib	486	−7.0	PI	no	0.21	1.1	0.01	12.9	16.5	nd	2	4.3	6.4	6	1	64	587	10.1
Sorafenib	465	−6.5	PI	yes	0.22	0.0	0.00	5.1	12.8	44	2	3.6	5.1	7	3	92	514	14.7
Sotorasib	561	−5.6	PI	yes	0.18	72.4	-0.72	11.0	31.5	nd	nd	3.5	4.4	9	1	104	697	16.6
Sunitinib	399	−2.9	S	no	0.50	97.8	0.98	3.9	11.8	nd	1	2.6	3.1	6	3	84	525	13.4
Talazoparib	380	−3.9	PI	yes	0.01	0.8	-0.01	4.5	6.3	nd	nd	2.6	2.3	7	3	89	393	14.1
Tepotinib	493	−5.2	nd	yes	nd	98.2	0.98	12.8	14.4	72	nd	3.4	4.1	8	0	97	635	15.4
Tivozanib	455	−6.0	PI	nd	0.01	14.5	-0.09	5.4	10.9	nd	nd	3.6	4.0	9	2	111	530	17.6
Tofacitinib	312	−2.2	VSS	yes	0.06	58.7	0.90	22.1	6.3	74	3	1.4	1.2	7	1	89	418	14.1
Trametinib	615	−7.6	PI	no	0.01	0.0	0.00	13.1	12.9	72	2	3.2	4.6	9	2	107	611	17.0
Tucatinib	481	−4.8	VSS	yes	2.08	52.4	0.54	10.6	10.2	nd	nd	3.1	3.9	10	2	114	567	18.1
Umbralisib	572	−3.6	PI	yes	0.17	0.5	0.01	8.0	12.1	nd	2	3.7	4.7	8	2	110	636	17.4
Upadacitinib	380	−3.8	VSS	yes	0.16	0.0	0.00	17.0	10.2	nd	nd	2.7	2.2	7	2	78	418	12.4
Vandetanib	475	−4.3	PI	yes	0.21	98.2	0.98	9.1	17.9	nd	2	3.6	4.3	6	1	63	530	10.0
Vemurafenib	490	−6.2	PI	yes	0.20	3.3	-0.03	3.5	14.5	nd	4	3.6	5.3	6	2	92	526	14.6
Vismodegib	421	−5.6	PI	yes	0.24	0.0	0.00	20.6	15.5	32	2	3.1	3.7	5	1	76	468	12.1
Zanubrutinib	472	−5.2	PI	yes	0.21	100.0	0.00	2.7	10.3	nd	nd	3.3	3.2	8	3	102	596	16.3
**Mean**	**473**	**−4.5**	**-**	**-**	**0.6**	**40.9**	**0.3**	**9.2**	**15.1**	**-**	**-**	**3.0**	**3.5**	**8**	**2**	**96**	**566**	**15.3**
**SD**	**70**	**1.3**	**-**	**-**	**1.1**	**42.9**	**0.5**	**7.1**	**12.7**	**-**	**-**	**0.7**	**1.2**	**2**	**1**	**21**	**97**	**3.4**

**Table 2:** Characteristics of principal component analysis (PCA). The left side of the table presents the relative contributions of the different variables to the main components (PC1 and PC3). The left side of the table presents the corresponding absolute values of coefficient of correlation indicating the strength of correlation.

	Contribution of Variables (%)		Absolute Value of Coefficient of Correlation	
	**F1**	**F3**	**F1**	**F3**
HBD	1.56	11.23	−0.240	−0.402
HBA	9.38	0.15	−0.588	−0.046
TPSA	19.25	2.63	−0.842	−0.195
%TPSA	20.00	5.79	−0.859	−0.289
LogS	10.45	3.54	−0.621	0.226
LogD	20.10	2.79	0.861	−0.200
LogP	16.14	0.67	0.771	−0.098
Caco-2 permeability	0.43	15.58	0.126	0.474
MDCK permeability	0.11	16.42	0.064	0.486
Fabs (%)	2.58	41.20	−0.308	0.770

## Data Availability

Not applicable.

## Supplementary Materials

The following supporting information can be downloaded at: https://www.mdpi.com/article/10.3390/pharmaceutics14112493/s1, Supplementary Materials include 3 Figures and 2 Tables. Figure S1. Algorithm in Python for the estimation of ka as a function of ke, t_max_. Figure S2. Distribution of the solubility at neutral pH classified according to the USP classification in a series of oral targeted anticancer drugs. Figure S3. Variations in MAT ratio following ±10% variations in t_max_ in a series of oral targeted anticancer drugs. Table S1. Pharmacologic class (ATC5 and ATC4 classification), initial approval indication and approval date by the FDA and the sponsor of oral targeted anticancer drugs. Table S2. Estimated concentration in the intestinal lumen at neutral pH (Igut, mM) for a series of oral targeted anticancer drugs.

## Abbreviations

ABC	ATP-binding cassette
ATC	anatomical therapeutic chemical
AUC	area under the curve
BCRP	breast cancer resistance protein
BCS	biopharmaceutics classification system
CL	clearance
DDI	drug–drug interaction
EMA	European Medicines Agency
ENT4	equilibrative nucleoside transporter
FDA	Food and Drug Administration
Fabs	absolute oral bioavailability
Fg	extent of the fraction of the dose not metabolized in the intestinal
Fh	extent of the fraction of the dose not metabolized in the liver
fm	fraction of the drug metabolized
HBD	hydrogen bond donor
HBA	hydrogen bond acceptor
IC_50_	half-maximal inhibitory concentration
Igut	concentration in the intestinal lumen at neutral pH
ITRA	itraconazole
IV	intravenous route
k_a_	rate of absorption
ke	apparent elimination rate
KETO	ketoconazole
Log D	logarithm of the distribution coefficient at pH 7.40
Log P	logarithm of the partition coefficient
Log S	logarithm of the solubility
MAT	mean absorption time
MATE	multidrug and toxin extrusion protein
MW	molecular weight
NDA	new drug application
OAT	organic anion transporter
OATP	organic anion transporting polypeptides transporter
OCT	organic cation transporter
OCTN	organic cation/carnitine transporter
PBPK	physiologically based pharmacokinetic
PCA	principal component analysis
P-gp	P-glycoprotein
PI	practically insoluble
PKI	protein kinase inhibitor
PMAT	plasma membrane monoamine transporter
POSA	posaconazole
PSA	polar surface area
RIF	rifampin
S	soluble
SLC	solute carriers
tmax	time of maximal plasma concentration
t1/2	apparent terminal half-life
τ	administration interval at steady state
TPSA	topological polar surface area
USP	United States Pharmacopeia
Vss	steady-state volume of distribution
VSS	very slightly soluble
