# Supplementary material for: Involvement of Transporters in Intestinal Drug–Drug Interactions of Oral Targeted Anticancer Drugs Assessed by Changes in Drug Absorption Time
Source: Pharmaceutics. 2022 Nov 17;14(11):2493. doi: 10.3390/pharmaceutics14112493 (PMC9696196; doi:10.3390/pharmaceutics14112493)
Supplement: Supplementary file 1 [file pharmaceutics-14-02493-s001.zip › Figure S3.pdf]

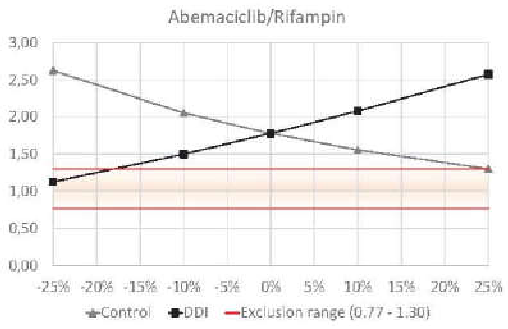
Abemaciclib/Rifampin
3,00
2,50
2,00
1,50
1,00
0,50
0,00
-25% -20% -15% -10% -5% 0% 5% 10% 15% 20% 25%
Control
DDI
Exclusion range (0.77 - 1.30)

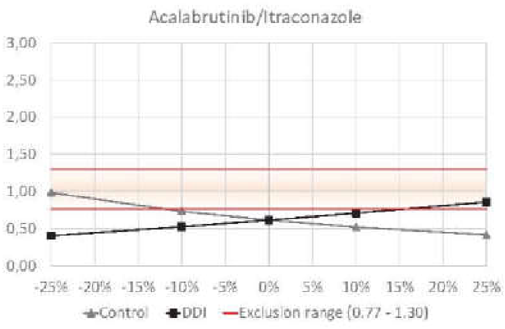
Acalabrutinib/Itraconazole
3,00
2,50
2,00
1,50
1,00
0,50
0,00
-25% -20% -15% -10% -5% 0% 5% 10% 15% 20% 25%
Control
DDI
Exclusion range (0.77 - 1.30)

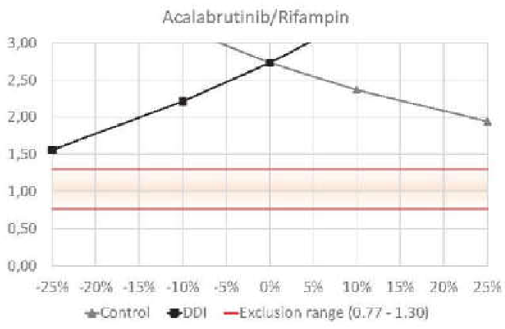
Acalabrutinib/Rifampin
3,00
2,50
2,00
1,50
1,00
0,50
0,00
-25% -20% -15% -10% -5% 0% 5% 10% 15% 20% 25%
Control
DDI
Exclusion range (0.77 - 1.30)

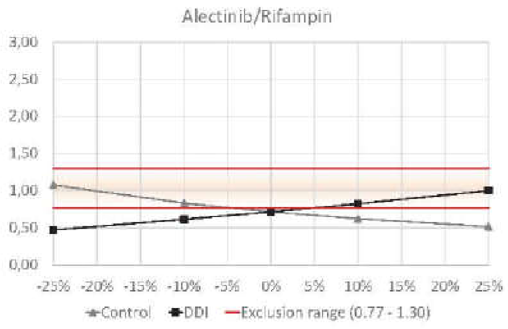
Alectinib/Rifampin
3,00
2,50
2,00
1,50
1,00
0,50
0,00
-25% -20% -15% -10% -5% 0% 5% 10% 15% 20% 25%
Control
DDI
Exclusion range (0.77 - 1.30)

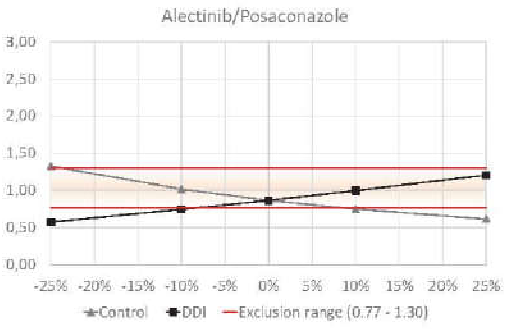
Alectinib/Posaconazole
3,00
2,50
2,00
1,50
1,00
0,50
0,00
-25% -20% -15% -10% -5% 0% 5% 10% 15% 20% 25%
Control
DDI
Exclusion range (0.77 - 1.30)

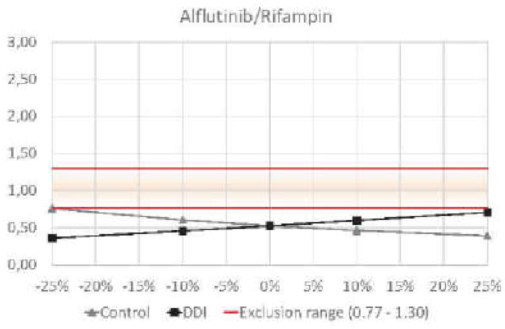
Alflutinib/Rifampin
3,00
2,50
2,00
1,50
1,00
0,50
0,00
-25% -20% -15% -10% -5% 0% 5% 10% 15% 20% 25%
Control
DDI
Exclusion range (0.77 - 1.30)

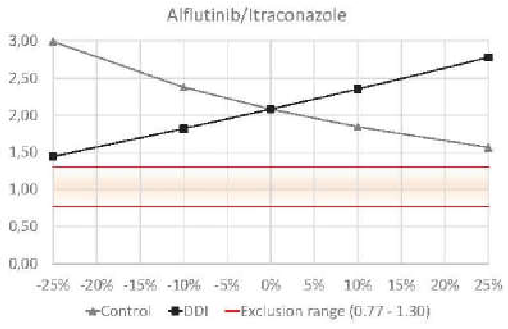
Alflutinib/Itraconazole
3,00
2,50
2,00
1,50
1,00
0,50
0,00
-25% -20% -15% -10% -5% 0% 5% 10% 15% 20% 25%
Control
DDI
Exclusion range (0.77 - 1.30)

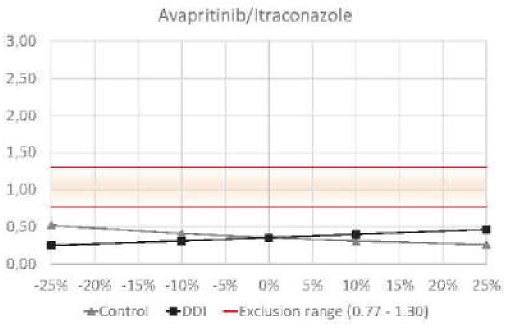
Avapritinib/Itraconazole
3,00
2,50
2,00
1,50
1,00
0,50
0,00
-25% -20% -15% -10% -5% 0% 5% 10% 15% 20% 25%
Control
DDI
Exclusion range (0.77 - 1.30)

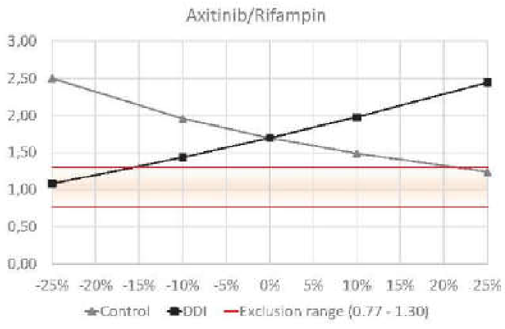
Axitinib/Rifampin
3,00
2,50
2,00
1,50
1,00
0,50
0,00
-25% -20% -15% -10% -5% 0% 5% 10% 15% 20% 25%
Control
DDI
Exclusion range (0.77 - 1.30)

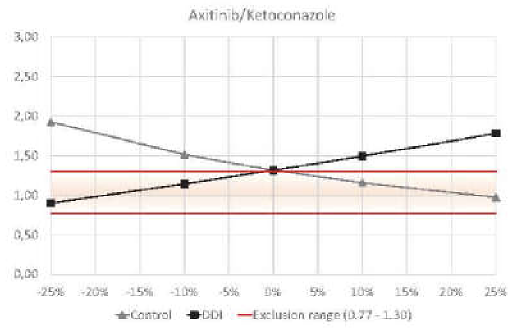
Axitinib/Ketoconazole
3,00 2,50 2,00 1,50 1,00 0,50 0,00
-25% -20% -15% -10% -5% 0% 5% 10% 15% 20% 25%
Control DDI Exclusion range (0.77 - 1.30)

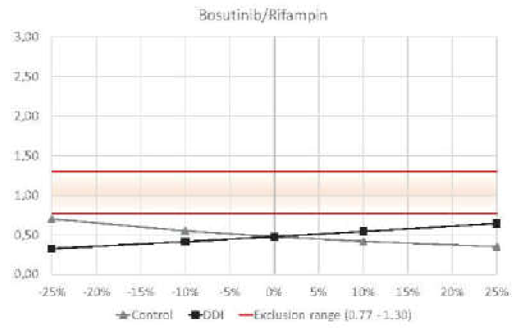
Bosutinib/Rifampin
3,00 2,50 2,00 1,50 1,00 0,50 0,00
-25% -20% -15% -10% -5% 0% 5% 10% 15% 20% 25%
Control DDI Exclusion range (0.77 - 1.30)

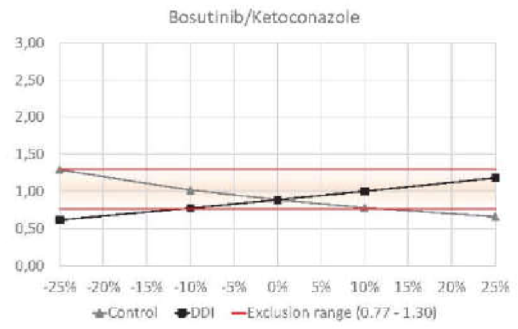
Bosutinib/Ketoconazole
3,00 2,50 2,00 1,50 1,00 0,50 0,00
-25% -20% -15% -10% -5% 0% 5% 10% 15% 20% 25%
Control DDI Exclusion range (0.77 - 1.30)

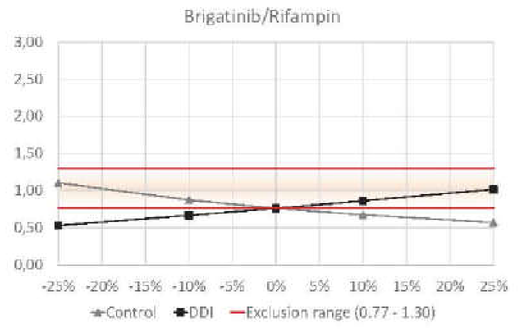
Brigatinib/Rifampin
3,00 2,50 2,00 1,50 1,00 0,50 0,00
-25% -20% -15% -10% -5% 0% 5% 10% 15% 20% 25%
Control DDI Exclusion range (0.77 - 1.30)

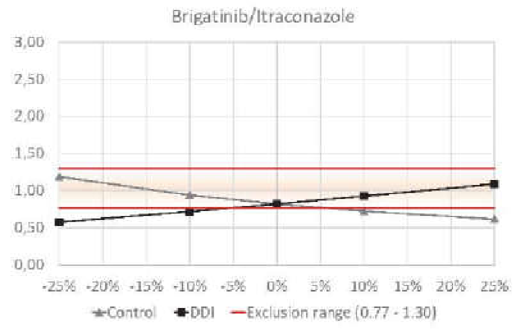
Brigatinib/Itraconazole
3,00 2,50 2,00 1,50 1,00 0,50 0,00
-25% -20% -15% -10% -5% 0% 5% 10% 15% 20% 25%
Control DDI Exclusion range (0.77 - 1.30)

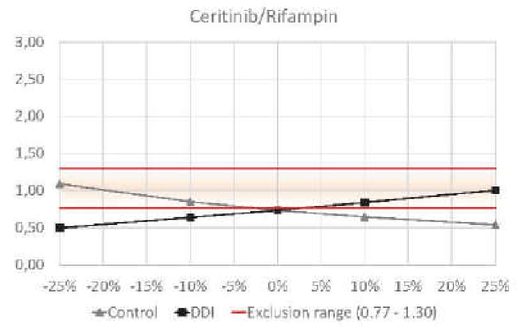
Ceritinib/Rifampin
3,00 2,50 2,00 1,50 1,00 0,50 0,00
-25% -20% -15% -10% -5% 0% 5% 10% 15% 20% 25%
Control DDI Exclusion range (0.77 - 1.30)

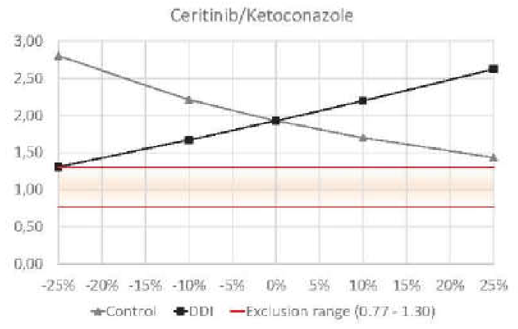
Ceritinib/Ketoconazole
3,00 2,50 2,00 1,50 1,00 0,50 0,00
-25% -20% -15% -10% -5% 0% 5% 10% 15% 20% 25%
Control DDI Exclusion range (0.77 - 1.30)

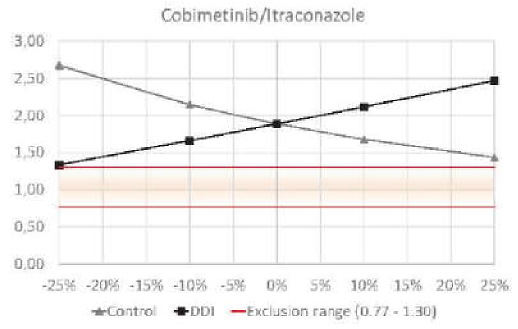
Cobimetinib/Itraconazole
3,00 2,50 2,00 1,50 1,00 0,50 0,00
-25% -20% -15% -10% -5% 0% 5% 10% 15% 20% 25%
Control DDI Exclusion range (0.77 - 1.30)

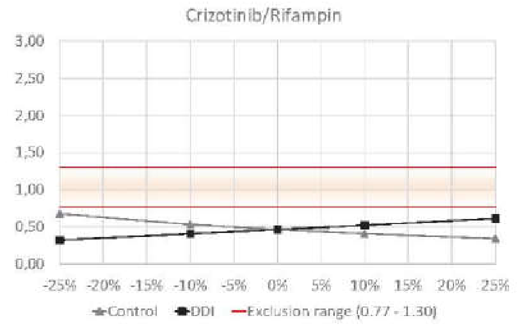
Crizotinib/Rifampin
3,00 2,50 2,00 1,50 1,00 0,50 0,00
-25% -20% -15% -10% -5% 0% 5% 10% 15% 20% 25%
Control DDI Exclusion range (0.77 - 1.30)

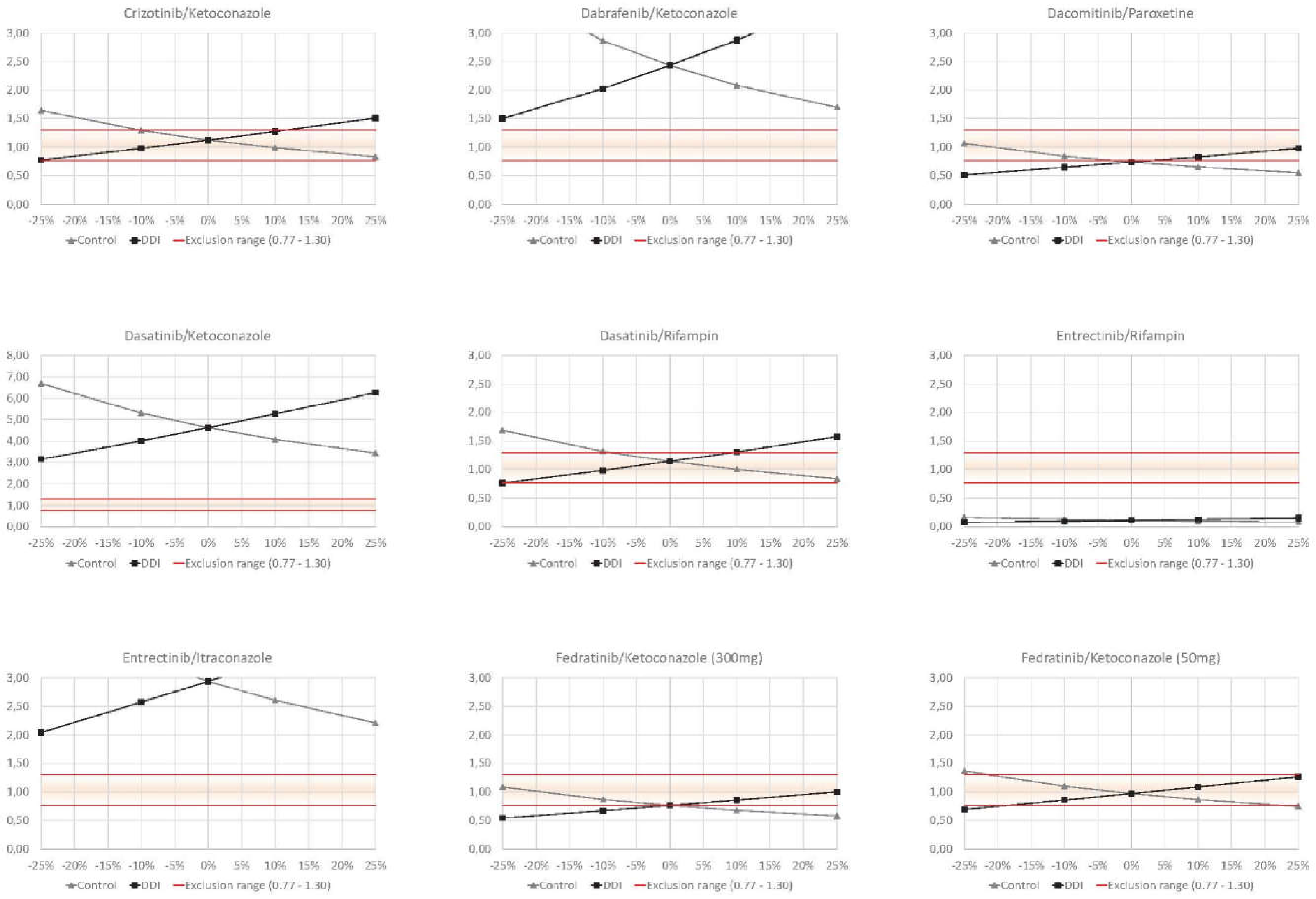
Crizotinib/Ketoconazole
Dabrafenib/Ketoconazole
Dacomitinib/Paroxetine
Dasatinib/Ketoconazole
Dasatinib/Rifampin
Entrectinib/Rifampin
Entrectinib/Itraconazole
Fedratinib/Ketoconazole (300mg)
Fedratinib/Ketoconazole (50mg)
0,00
0,50
1,00
1,50
2,00
2,50
3,00
4,00
5,00
6,00
7,00
8,00
-25%
-20%
-15%
-10%
-5%
0%
5%
10%
15%
20%
25%
Control
DDI
Exclusion range (0.77 - 1.30)

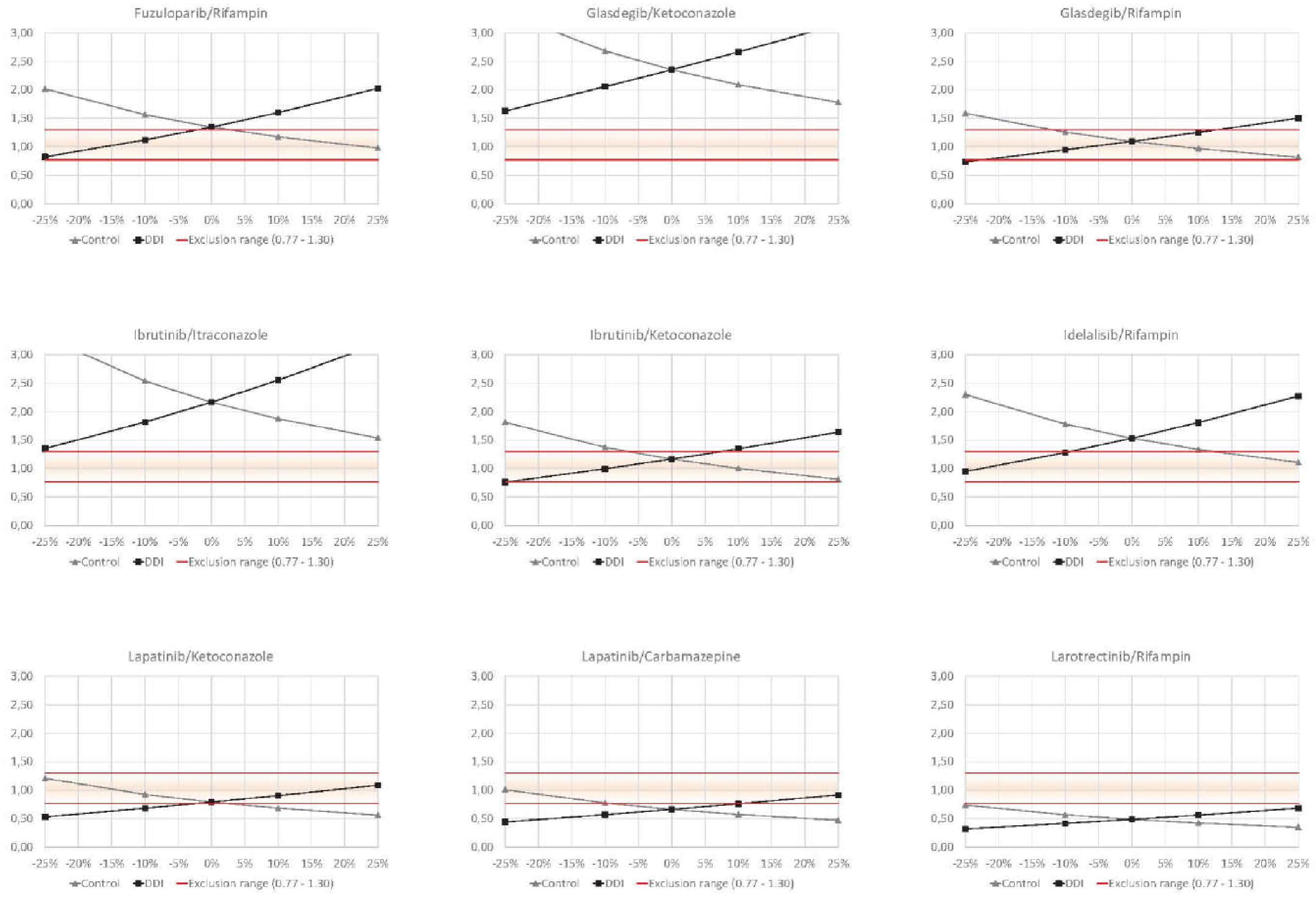

Fuzuloparib/Rifampin
Glasdegib/Ketoconazole
Glasdegib/Rifampin
Ibrutinib/Itraconazole
Ibrutinib/Ketoconazole
Idelalisib/Rifampin
Lapatinib/Ketoconazole
Lapatinib/Carbamazepine
Larotrectinib/Rifampin
0,00 0,50 1,00 1,50 2,00 2,50 3,00
-25% -20% -15% -10% -5% 0% 5% 10% 15% 20% 25%
Control
DDI
Exclusion range (0.77 - 1.30)

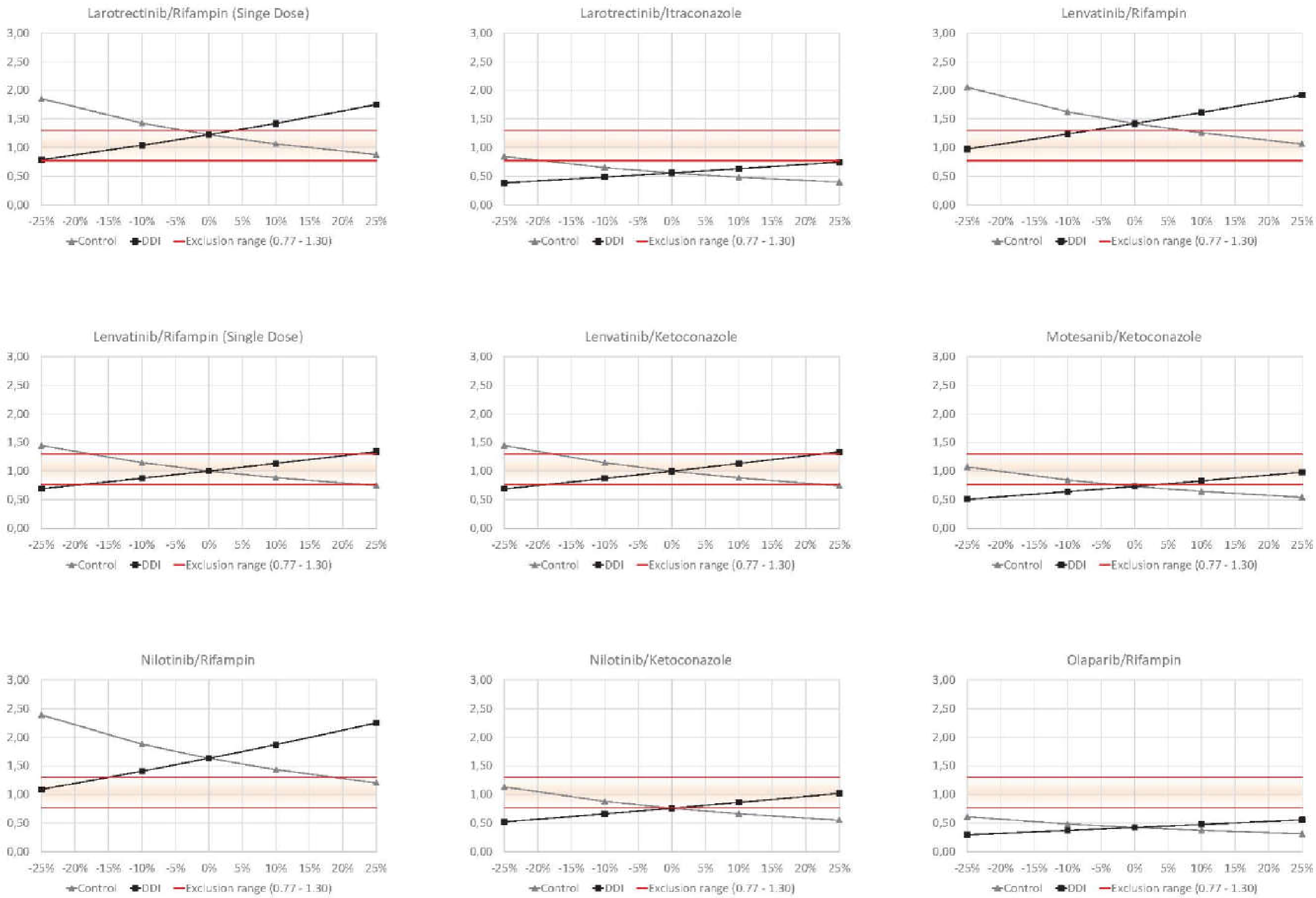
Larotrectinib/Rifampin (Singe Dose)
Larotrectinib/Itraconazole
Lenvatinib/Rifampin
Lenvatinib/Rifampin (Single Dose)
Lenvatinib/Ketoconazole
Motesanib/Ketoconazole
Nilotinib/Rifampin
Nilotinib/Ketoconazole
Olaparib/Rifampin
0,00
0,50
1,00
1,50
2,00
2,50
3,00
-25% -20% -15% -10% -5% 0% 5% 10% 15% 20% 25%
Control
DDI
Exclusion range (0.77 - 1.30)

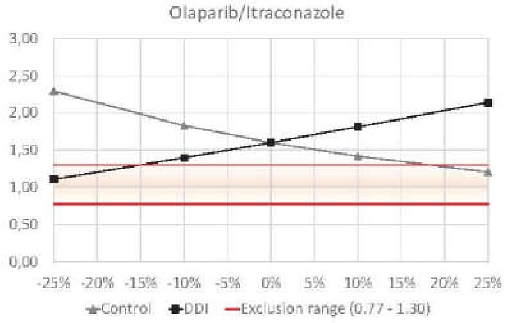
Olaparib/Itraconazole
3,00
2,50
2,00
1,50
1,00
0,50
0,00
-25% -20% -15% -10% -5% 0% 5% 10% 15% 20% 25%
Control
DDI
Exclusion range (0.77 - 1.30)

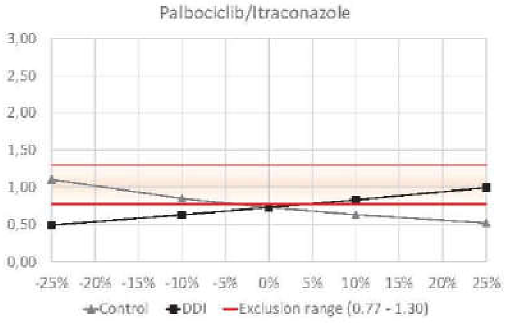
Palbociclib/Itraconazole
3,00
2,50
2,00
1,50
1,00
0,50
0,00
-25% -20% -15% -10% -5% 0% 5% 10% 15% 20% 25%
Control
DDI
Exclusion range (0.77 - 1.30)

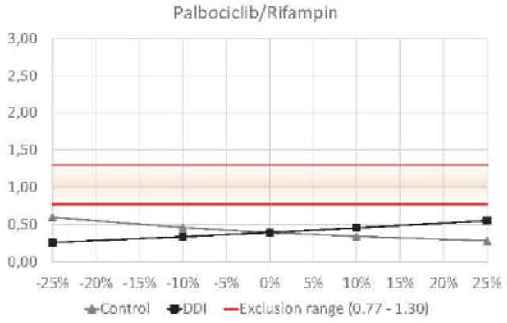
Palbociclib/Rifampin
3,00
2,50
2,00
1,50
1,00
0,50
0,00
-25% -20% -15% -10% -5% 0% 5% 10% 15% 20% 25%
Control
DDI
Exclusion range (0.77 - 1.30)

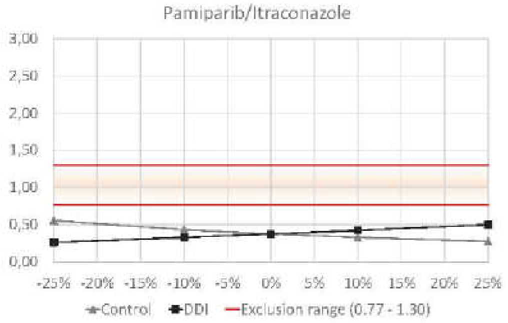
Pamiparib/Itraconazole
3,00
2,50
2,00
1,50
1,00
0,50
0,00
-25% -20% -15% -10% -5% 0% 5% 10% 15% 20% 25%
Control
DDI
Exclusion range (0.77 - 1.30)

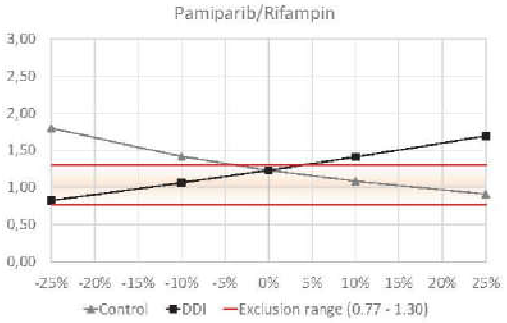
Pamiparib/Rifampin
3,00
2,50
2,00
1,50
1,00
0,50
0,00
-25% -20% -15% -10% -5% 0% 5% 10% 15% 20% 25%
Control
DDI
Exclusion range (0.77 - 1.30)

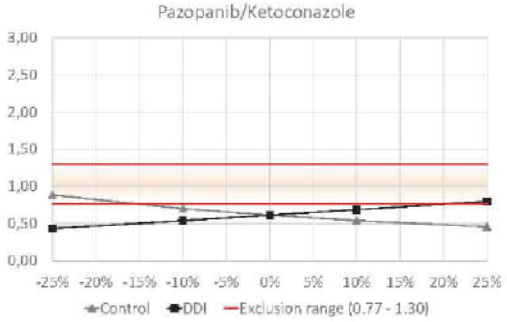
Pazopanib/Ketoconazole
3,00
2,50
2,00
1,50
1,00
0,50
0,00
-25% -20% -15% -10% -5% 0% 5% 10% 15% 20% 25%
Control
DDI
Exclusion range (0.77 - 1.30)

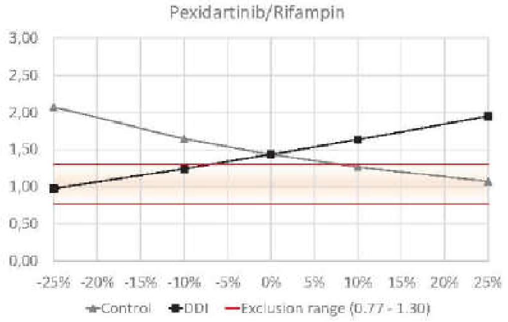
Pexidartinib/Rifampin
3,00
2,50
2,00
1,50
1,00
0,50
0,00
-25% -20% -15% -10% -5% 0% 5% 10% 15% 20% 25%
Control
DDI
Exclusion range (0.77 - 1.30)

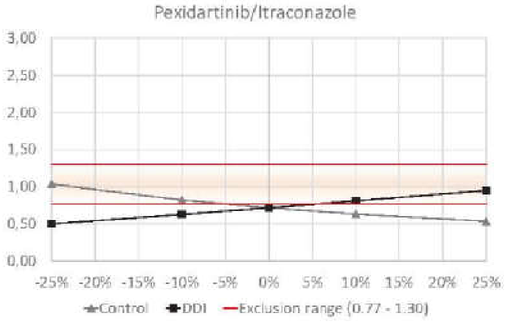
Pexidartinib/Itraconazole
3,00
2,50
2,00
1,50
1,00
0,50
0,00
-25% -20% -15% -10% -5% 0% 5% 10% 15% 20% 25%
Control
DDI
Exclusion range (0.77 - 1.30)

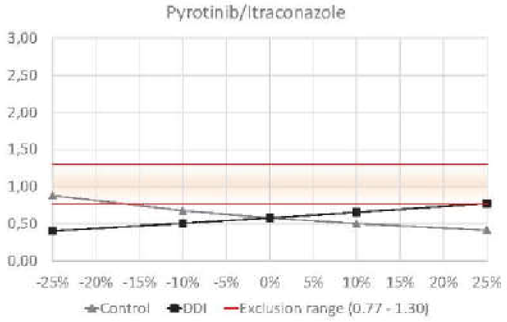
Pyrotinib/Itraconazole
3,00
2,50
2,00
1,50
1,00
0,50
0,00
-25% -20% -15% -10% -5% 0% 5% 10% 15% 20% 25%
Control
DDI
Exclusion range (0.77 - 1.30)

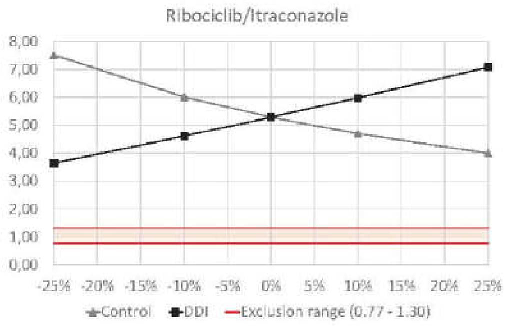
Ribociclib/Itraconazole
Control
DDI
Exclusion range (0.77 - 1.30)

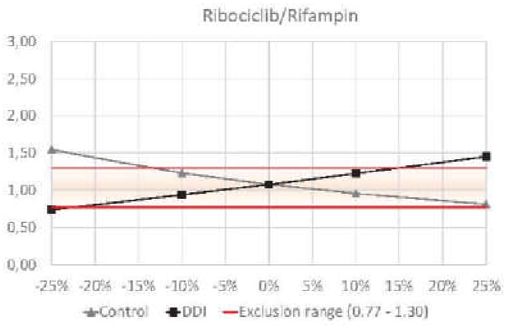
Ribociclib/Rifampin
Control
DDI
Exclusion range (0.77 - 1.30)

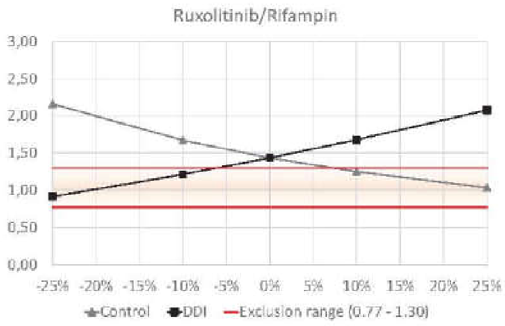
Ruxolitinib/Rifampin
Control
DDI
Exclusion range (0.77 - 1.30)

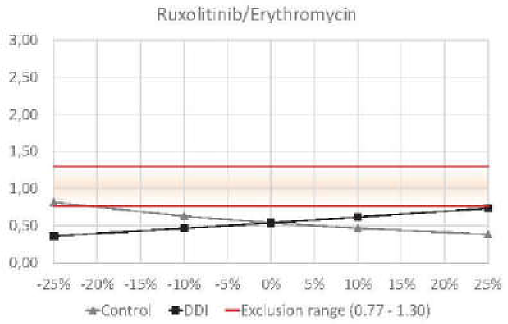
Ruxolitinib/Erythromycin
Control
DDI
Exclusion range (0.77 - 1.30)

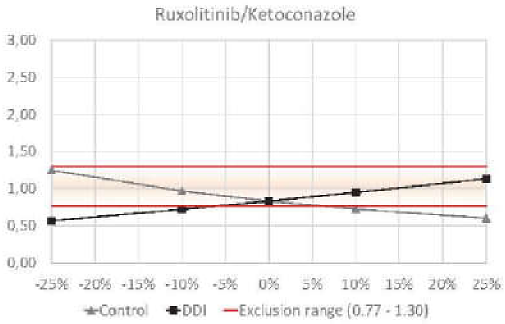
Ruxolitinib/Ketoconazole
Control
DDI
Exclusion range (0.77 - 1.30)

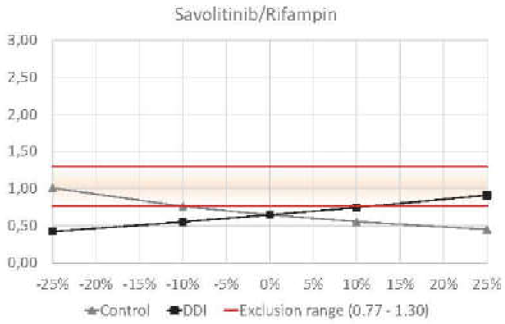
Savolitinib/Rifampin
Control
DDI
Exclusion range (0.77 - 1.30)

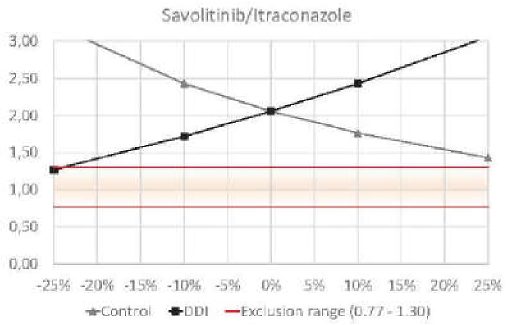
Savolitinib/Itraconazole
Control
DDI
Exclusion range (0.77 - 1.30)

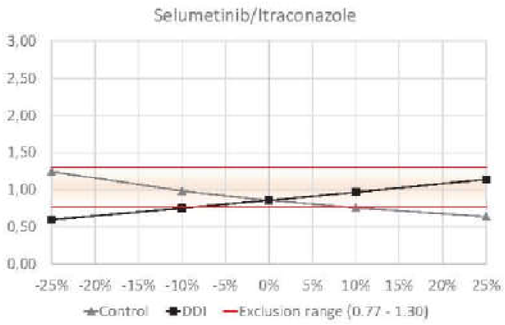
Selumetinib/Itraconazole
Control
DDI
Exclusion range (0.77 - 1.30)

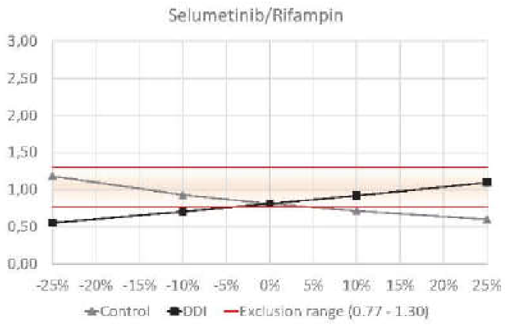
Selumetinib/Rifampin
Control
DDI
Exclusion range (0.77 - 1.30)

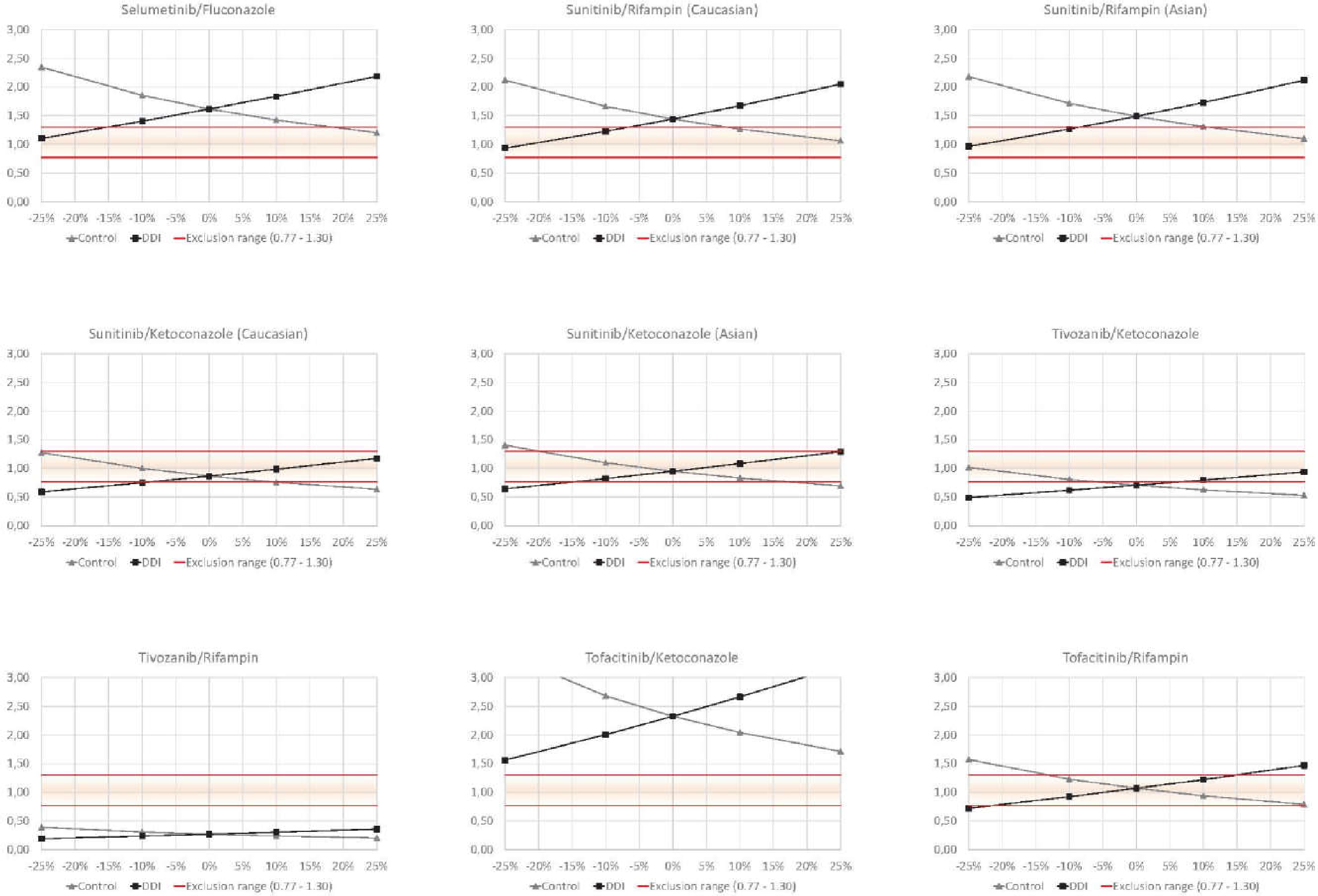
Selumetinib/Fluconazole
Sunitinib/Rifampin (Caucasian)
Sunitinib/Rifampin (Asian)
Sunitinib/Ketoconazole (Caucasian)
Sunitinib/Ketoconazole (Asian)
Tivozanib/Ketoconazole
Tivozanib/Rifampin
Tofacitinib/Ketoconazole
Tofacitinib/Rifampin
3,00 2,50 2,00 1,50 1,00 0,50 0,00
-25% -20% -15% -10% -5% 0% 5% 10% 15% 20% 25%
Control DDI Exclusion range (0.77 - 1.30)

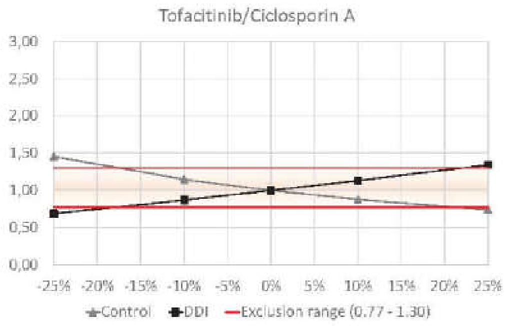
Tofacitinib/Ciclosporin A
3,00 2,50 2,00 1,50 1,00 0,50 0,00
-25% -20% -15% -10% -5% 0% 5% 10% 15% 20% 25%
Control DDI Exclusion range (0.77 - 1.30)

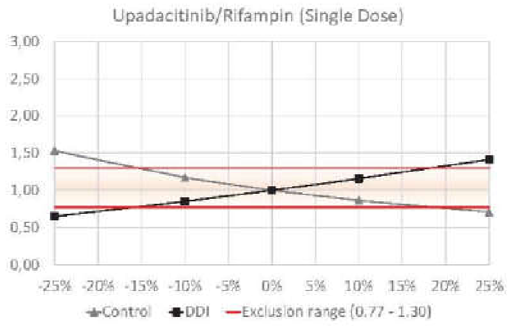
Upadacitinib/Rifampin (Single Dose)
3,00 2,50 2,00 1,50 1,00 0,50 0,00
-25% -20% -15% -10% -5% 0% 5% 10% 15% 20% 25%
Control DDI Exclusion range (0.77 - 1.30)

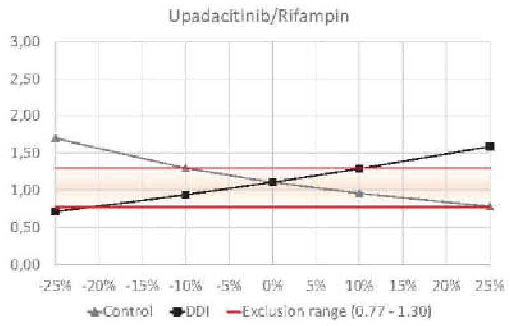
Upadacitinib/Rifampin
3,00 2,50 2,00 1,50 1,00 0,50 0,00
-25% -20% -15% -10% -5% 0% 5% 10% 15% 20% 25%
Control DDI Exclusion range (0.77 - 1.30)

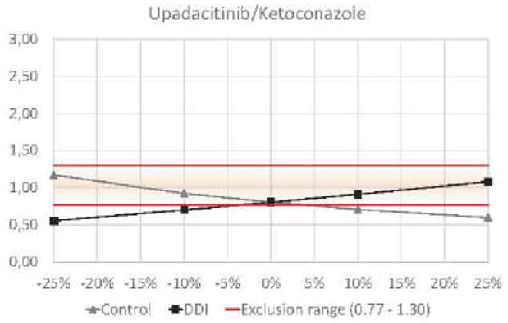
Upadacitinib/Ketoconazole
3,00 2,50 2,00 1,50 1,00 0,50 0,00
-25% -20% -15% -10% -5% 0% 5% 10% 15% 20% 25%
Control DDI Exclusion range (0.77 - 1.30)

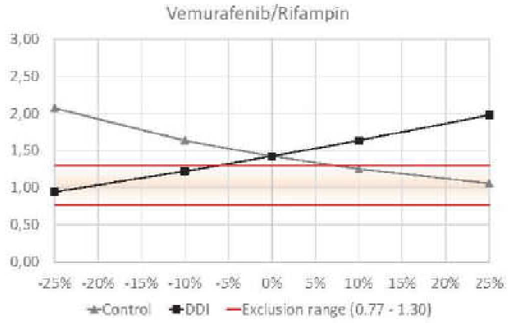
Vemurafenib/Rifampin
3,00 2,50 2,00 1,50 1,00 0,50 0,00
-25% -20% -15% -10% -5% 0% 5% 10% 15% 20% 25%
Control DDI Exclusion range (0.77 - 1.30)

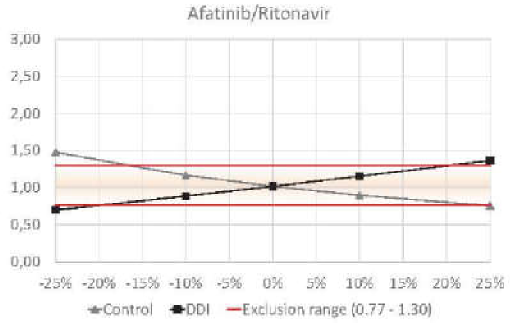
Afatinib/Ritonavir
3,00 2,50 2,00 1,50 1,00 0,50 0,00
-25% -20% -15% -10% -5% 0% 5% 10% 15% 20% 25%
Control DDI Exclusion range (0.77 - 1.30)

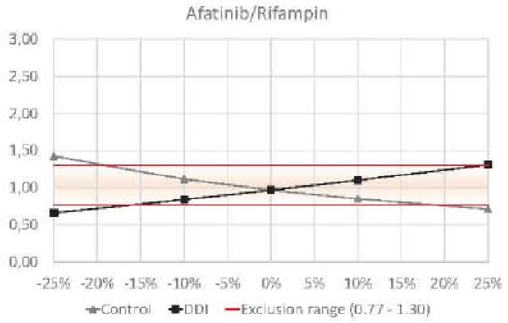
Afatinib/Rifampin
3,00 2,50 2,00 1,50 1,00 0,50 0,00
-25% -20% -15% -10% -5% 0% 5% 10% 15% 20% 25%
Control DDI Exclusion range (0.77 - 1.30)

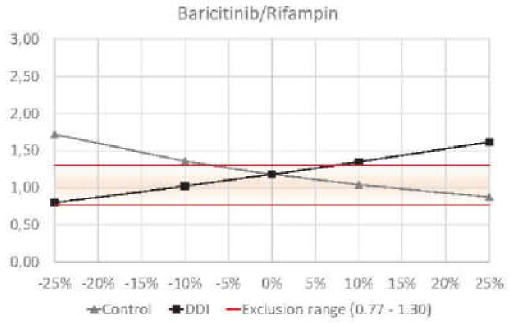
Baricitinib/Rifampin
3,00 2,50 2,00 1,50 1,00 0,50 0,00
-25% -20% -15% -10% -5% 0% 5% 10% 15% 20% 25%
Control DDI Exclusion range (0.77 - 1.30)

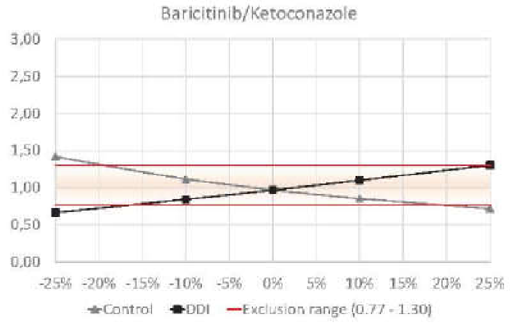
Baricitinib/Ketoconazole
3,00 2,50 2,00 1,50 1,00 0,50 0,00
-25% -20% -15% -10% -5% 0% 5% 10% 15% 20% 25%
Control DDI Exclusion range (0.77 - 1.30)

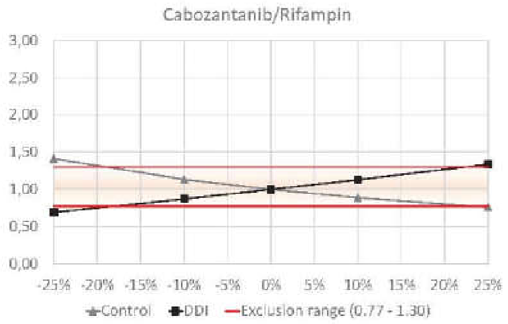
Cabozantanib/Rifampin
3,00
2,50
2,00
1,50
1,00
0,50
0,00
-25% -20% -15% -10% -5% 0% 5% 10% 15% 20% 25%
Control
DDI
Exclusion range (0.77 - 1.30)

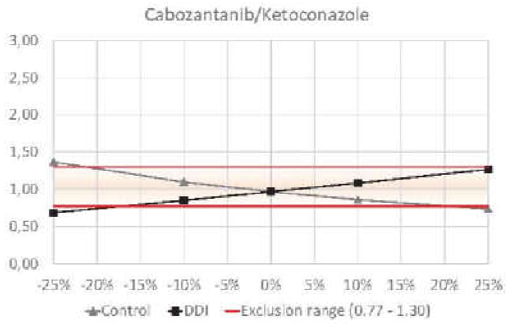
Cabozantanib/Ketoconazole
3,00
2,50
2,00
1,50
1,00
0,50
0,00
-25% -20% -15% -10% -5% 0% 5% 10% 15% 20% 25%
Control
DDI
Exclusion range (0.77 - 1.30)

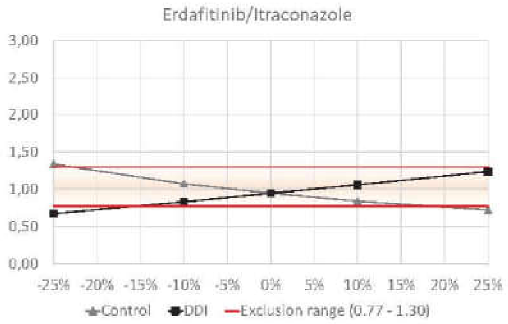
Erdafitinib/Itraconazole
3,00
2,50
2,00
1,50
1,00
0,50
0,00
-25% -20% -15% -10% -5% 0% 5% 10% 15% 20% 25%
Control
DDI
Exclusion range (0.77 - 1.30)

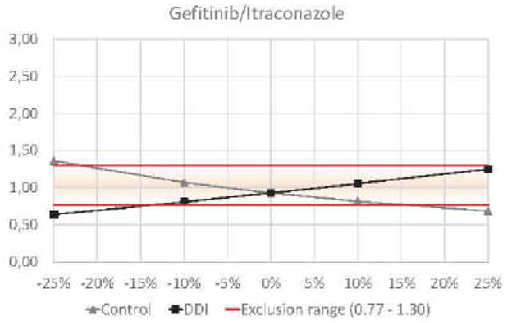
Gefitinib/Itraconazole
3,00
2,50
2,00
1,50
1,00
0,50
0,00
-25% -20% -15% -10% -5% 0% 5% 10% 15% 20% 25%
Control
DDI
Exclusion range (0.77 - 1.30)

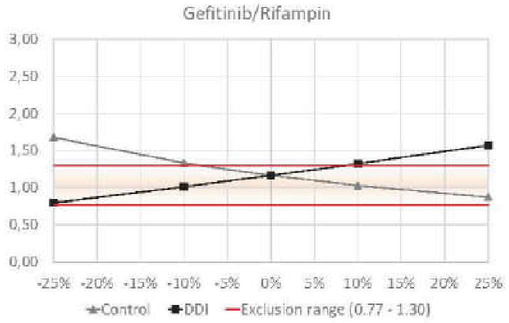
Gefitinib/Rifampin
3,00
2,50
2,00
1,50
1,00
0,50
0,00
-25% -20% -15% -10% -5% 0% 5% 10% 15% 20% 25%
Control
DDI
Exclusion range (0.77 - 1.30)

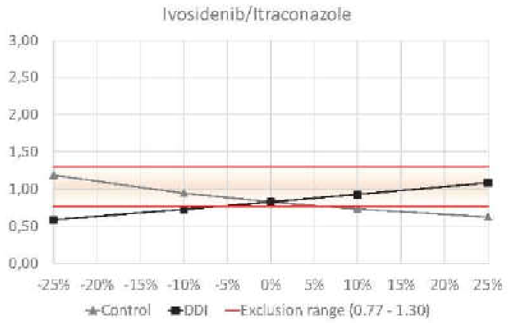
Ivosidenib/Itraconazole
3,00
2,50
2,00
1,50
1,00
0,50
0,00
-25% -20% -15% -10% -5% 0% 5% 10% 15% 20% 25%
Control
DDI
Exclusion range (0.77 - 1.30)

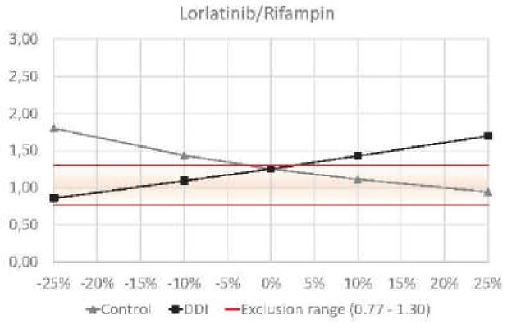
Lorlatinib/Rifampin
3,00
2,50
2,00
1,50
1,00
0,50
0,00
-25% -20% -15% -10% -5% 0% 5% 10% 15% 20% 25%
Control
DDI
Exclusion range (0.77 - 1.30)

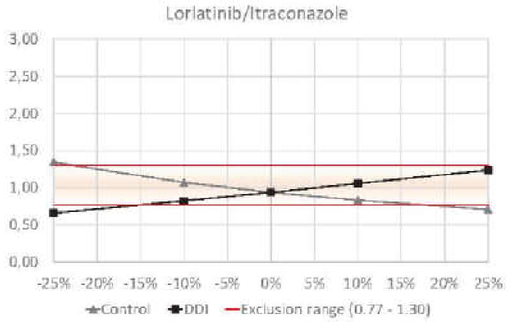
Lorlatinib/Itraconazole
3,00
2,50
2,00
1,50
1,00
0,50
0,00
-25% -20% -15% -10% -5% 0% 5% 10% 15% 20% 25%
Control
DDI
Exclusion range (0.77 - 1.30)

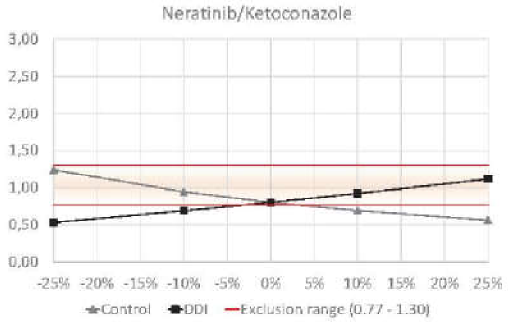
Neratinib/Ketoconazole
3,00
2,50
2,00
1,50
1,00
0,50
0,00
-25% -20% -15% -10% -5% 0% 5% 10% 15% 20% 25%
Control
DDI
Exclusion range (0.77 - 1.30)

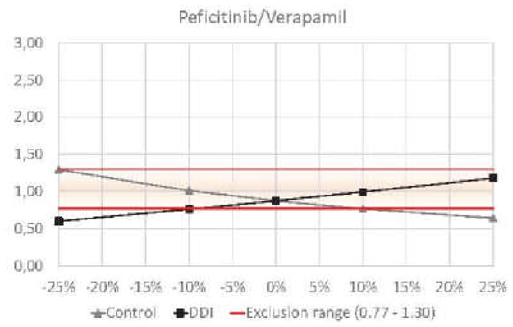
Peficitinib/Verapamil
3,00
2,50
2,00
1,50
1,00
0,50
0,00
-25% -20% -15% -10% -5% 0% 5% 10% 15% 20% 25%
Control
DDI
Exclusion range (0.77 - 1.30)

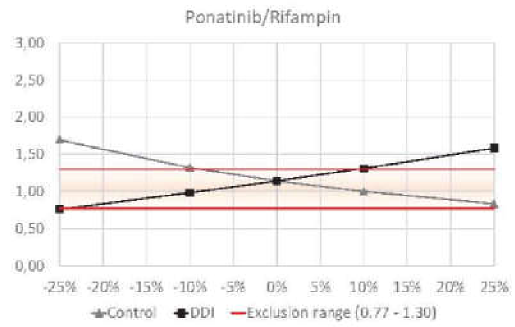
Ponatinib/Rifampin
3,00
2,50
2,00
1,50
1,00
0,50
0,00
-25% -20% -15% -10% -5% 0% 5% 10% 15% 20% 25%
Control
DDI
Exclusion range (0.77 - 1.30)

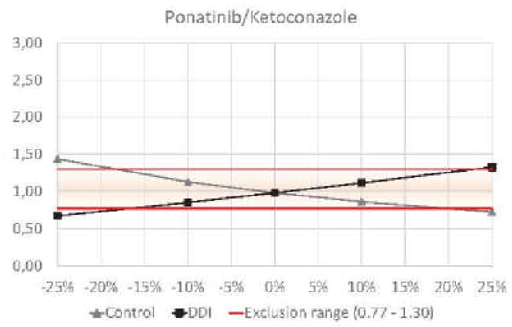
Ponatinib/Ketoconazole
3,00
2,50
2,00
1,50
1,00
0,50
0,00
-25% -20% -15% -10% -5% 0% 5% 10% 15% 20% 25%
Control
DDI
Exclusion range (0.77 - 1.30)

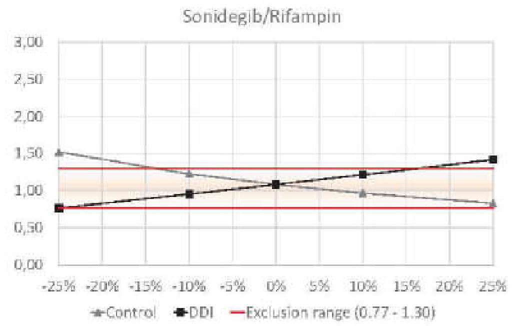
Sonidegib/Rifampin
3,00
2,50
2,00
1,50
1,00
0,50
0,00
-25% -20% -15% -10% -5% 0% 5% 10% 15% 20% 25%
Control
DDI
Exclusion range (0.77 - 1.30)

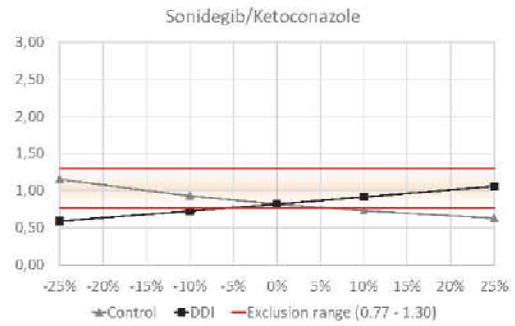
Sonidegib/Ketoconazole
3,00
2,50
2,00
1,50
1,00
0,50
0,00
-25% -20% -15% -10% -5% 0% 5% 10% 15% 20% 25%
Control
DDI
Exclusion range (0.77 - 1.30)

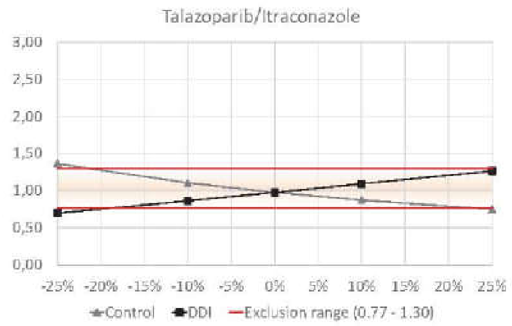
Talazoparib/Itraconazole
3,00
2,50
2,00
1,50
1,00
0,50
0,00
-25% -20% -15% -10% -5% 0% 5% 10% 15% 20% 25%
Control
DDI
Exclusion range (0.77 - 1.30)

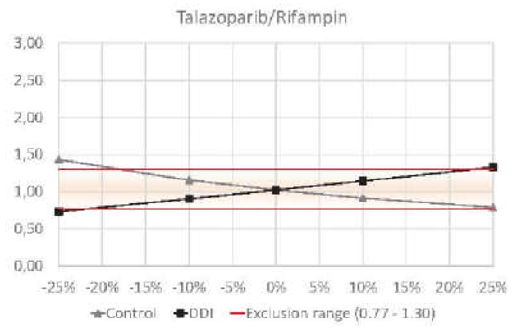
Talazoparib/Rifampin
3,00
2,50
2,00
1,50
1,00
0,50
0,00
-25% -20% -15% -10% -5% 0% 5% 10% 15% 20% 25%
Control
DDI
Exclusion range (0.77 - 1.30)

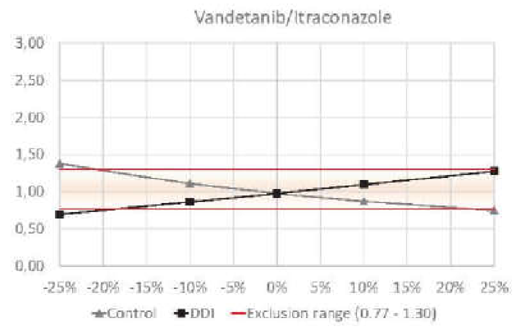
Vandetanib/Itraconazole
3,00
2,50
2,00
1,50
1,00
0,50
0,00
-25% -20% -15% -10% -5% 0% 5% 10% 15% 20% 25%
Control
DDI
Exclusion range (0.77 - 1.30)

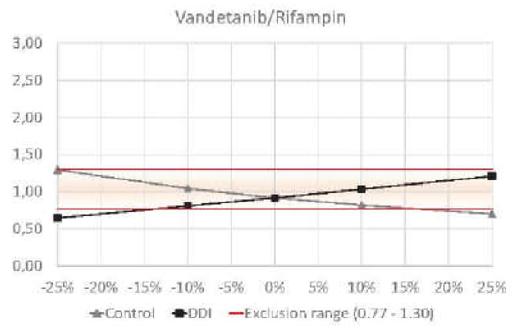
Vandetanib/Rifampin
3,00
2,50
2,00
1,50
1,00
0,50
0,00
-25% -20% -15% -10% -5% 0% 5% 10% 15% 20% 25%
Control
DDI
Exclusion range (0.77 - 1.30)

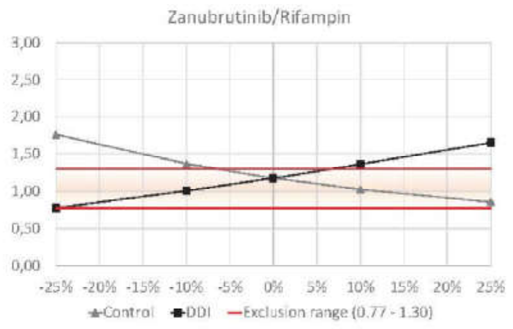

Zanubrutinib/Rifampin
3,00
2,50
2,00
1,50
1,00
0,50
0,00
-25% -20% -15% -10% -5% 0% 5% 10% 15% 20% 25%
Control
DDI
Exclusion range (0.77 - 1.30)

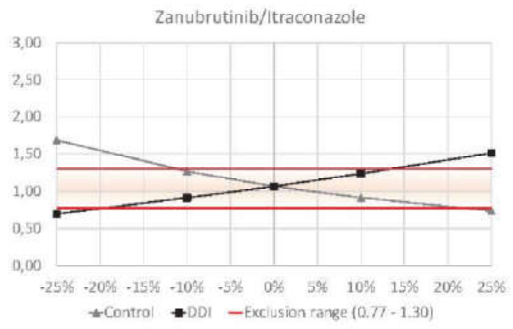

Zanubrutinib/Itraconazole
3,00
2,50
2,00
1,50
1,00
0,50
0,00
-25% -20% -15% -10% -5% 0% 5% 10% 15% 20% 25%
Control
DDI
Exclusion range (0.77 - 1.30)

Annex 3. Simulations in MAT ratio (y-axis) following ± 10 % and ± 25% variations in Tmax (x-axis) in a series of oral targeted anticancer drugs. The calculated MAT ratio from the respective induction and inhibition DDI studies correspond to 0 %.

MAT ratio outside the 0.77-1.30 interval, and remaining outside this interval following simulation variations in Tmax (± 10 %) are considered as relevant marker of a variation in the absorption rate. The methodology of calculation is described in details in the material and method section.
