# Supplementary material for: Involvement of Transporters in Intestinal Drug–Drug Interactions of Oral Targeted Anticancer Drugs Assessed by Changes in Drug Absorption Time
Source: Pharmaceutics. 2022 Nov 17;14(11):2493. doi: 10.3390/pharmaceutics14112493 (PMC9696196; doi:10.3390/pharmaceutics14112493)
Supplement: Supplementary file 1 [file pharmaceutics-14-02493-s001.zip › Table S1.pdf]

Table S1. Pharmacologic class (ATC5 and ATC4 classification), initial approval indication and approval date by the FDA and the sponsor of oral targeted anticancer drugs.

Drug	ATC5 classification	ATC4 classification	Initial approval indication	FDA Approval	Sponsor
Abemaciclib	Cyclin-dependent kinase (cdk) inhibitors	kinase inhibitor	breast cancer	2017	Eli Lilly
Acalabrutinib	Bruton's tyrosine kinase inhibitor	kinase inhibitor	mantle cell lymphoma	2017	Acerta Pharma B.V.
Afatinib	Epidermal growth factor receptor (egfr) tyrosine kinase inhibitors	kinase inhibitor	non-small cell lung cancer	2013	Boehringer Ingelheim
Alectinib	Anaplastic lymphoma kinase (alk) inhibitors	kinase inhibitor	non-small cell lung cancer	2016	Roche
Alpelisib	Phosphatidylinositol-3-kinase (pi3k) inhibitor	kinase inhibitor	breast cancer	2019	Novartis
Avapritinib	Other protein kinase inhibitors	kinase inhibitor	gastrointestinal stromal tumor	2020	Blueprint Medicines Corporation
Axitinib	Vascular endothelial growth factor receptor (vegfr) tyrosine kinase inhibitors	kinase inhibitor	renal cell carcinoma	2012	Pfizer
Baricitinib	Selective immunosuppressants	kinase inhibitor	rheumatoid arthritis	2018	Eli Lilly
Binimetinib	Mitogen-activated protein kinase (mek) inhibitor	kinase inhibitor	melanoma	2018	Array Biopharma
Bosutinib	Bcr-abl tyrosine kinase inhibitor	kinase inhibitor	chronic myelogenous leukemia	2012	Pfizer
Brigatinib	Anaplastic lymphoma kinase (alk) inhibitors	kinase inhibitor	non-small cell lung cancer	2017	Ariad
Cabozantinib	Other protein kinase inhibitors	kinase inhibitor	medullary thyroid cancer	2012	Exilixis
Capmatinib	Other protein kinase inhibitors	kinase inhibitor	non-small cell lung cancer	2020	Novartis
Ceritinib	Anaplastic lymphoma kinase (alk) inhibitors	kinase inhibitor	non-small cell lung cancer	2014	Novartis
Cobimetinib	Mitogen-activated protein kinase (mek) inhibitors	kinase inhibitor	melanoma	2015	Genentech
Crizotinib	Anaplastic lymphoma kinase (alk) inhibitors	kinase inhibitor	non-small cell lung cancer	2011	Pfizer
Dabrafenib	B-raf serine-threonine kinase (braf) inhibitors	kinase inhibitor	melanoma	2013	GSK
Dacomitinib	Epidermal growth factor receptor (egfr) tyrosine kinase inhibitors	kinase inhibitor	non-small cell lung cancer	2018	Pfizer
Dasatinib	Tyrosine kinase inhibitor	kinase inhibitor	chronic myelogenous leukemia	2006	BMS
Duvelisib	Kinase inhibitor - dual inhibitor of PI3K-δ and PI3K-γ	kinase inhibitor	chronic Lymphocytic Leukemia & Small Lymphocytic Lymphoma	2018	Verastem
Enasidenib	Other antineoplastic agents	IDH-I	acute myeloid leukemia	2017	Celgene
Encorafenib	B-raf serine-threonine kinase (braf) inhibitors	kinase inhibitor	melanoma	2018	Array
Entrectinib	Other protein kinase inhibitors	kinase inhibitor	non-small cell lung cancer	2019	Genentech
Erdafitinib	Kinase inhibitor	kinase inhibitor	urothelial carcinoma	2019	Janssen
Erlotinib	Epidermal growth factor receptor (egfr) tyrosine kinase inhibitors	kinase inhibitor	non-small cell lung cancer	2005	Genentech
Fedratinib	Janus-associated kinase (jak) inhibitors	kinase inhibitor	myelofibrosis	2019	Celgene
Fostamatinib	Other systemic hemostatics (Syk kinase inhibitor)	kinase inhibitor	chronic immune thrombocytopenia	2018	Rigel Pharmaceuticals
Gefitinib	tyrosine kinase inhibitor	kinase inhibitor	non-small cell lung cancer	2003	Astra Zeneca
Gilteritinib	Other protein kinase inhibitors	kinase inhibitor	acute myeloid leukemia	2018	Astellas
Glasdegib	Hedgehog pathway inhibitors	HP-I	acute myeloid leukemia	2018	Pfizer
Ibrutinib	Inhibitor of Bruton's tyrosine kinase	kinase inhibitor	chronic lymphocytic leukemia and Mantle cell lymphoma	2013	Pharmacyclics,
Idelalisib	Inhibitor of phosphatidylinositol 3-kinase, PI3Kδ	kinase inhibitor	chronic lymphocytic leukemia, follicular B-cell non-Hodgkin lymphoma and small lymphocytic	2014	Gilead
Imatinib	tyrosine kinase inhibitor	kinase inhibitor	chronic myelogenous leukemia	2001	Novartis
Infigratinib	tyrosine kinase inhibitor	kinase inhibitor	cholangiocarcinoma	2021	QED Therapeutics
Ivosidenib	Other antineoplastic agents	IDH-I	acute myeloid leukemia	2018	Agios Pharmaceuticals
Lapatinib	Kinase inhibitor	kinase inhibitor	breast cancer	2007	Novartis
Larotrectinib	Other protein kinase inhibitors	kinase inhibitor	solid tumors harboring an NTRK gene fusion	2018	Bayer - Loxo Oncology
Lenvatinib	Other protein kinase inhibitors	kinase inhibitor	thyroid cancer, renal cell carcinoma, hepatocellular carcinoma and	2015	Eisai
Lorlatinib	Anaplastic lymphoma kinase (alk) inhibitors	kinase inhibitor	non-small cell lung cancer	2018	Pfizer
Midostaurin	Other protein kinase inhibitors	kinase inhibitor	acute myeloid leukemia, systemic mastocytosis and mast cell leukemia	2017	Novartis
Neratinib	Human epidermal growth factor receptor 2 (her2) tyrosine kinase inhibitors	kinase inhibitor	breast cancer	2017	Puma Biotechnology
Nilotinib	tyrosine kinase inhibitor	kinase inhibitor	chronic myeloid leukemia	2007	Novartis
Niraparib	poly(ADP-ribose) polymerase (PARP) inhibitors	PARP-I	ovarian, fallopian tube, or primary peritoneal cancer	2017	Tesaro
Olaparib	poly(ADP-ribose) polymerase (PARP) inhibitors	PARP-I	ovarian, breast, pancreatic and prostate cancer	2017	Astra Zeneca
Osimertinib	Kinase inhibitor	kinase inhibitor	non-small cell lung cancer	2015	Astra Zeneca
Palbociclib	Cyclin-dependent kinase (cdk) inhibitors	kinase inhibitor	breast cancer	2015	Pfizer
Pazopanib	tyrosine kinase inhibitor	kinase inhibitor	renal cell carcinoma	2009	GSK
Pemigatinib	Kinase inhibitor	kinase inhibitor	cholangiocarcinoma	2020	Incyte Corporation
Pexidartinib	Kinase inhibitor	kinase inhibitor	tenosynovial giant cell tumor	2019	Daiichi Sankyo
Ponatinib	tyrosine kinase inhibitor	kinase inhibitor	chronic myelogenous leukemia and acute lymphoblastic leukemia	2012	Ariad
Pralsetinib	Kinase inhibitor	kinase inhibitor	non-small cell lung cancer	2020	Blueprint Medicines Corporation
Regorafenib	Other protein kinase inhibitors	kinase inhibitor	colorectal cancer	2012	Bayer
Ribociclib	Cyclin-dependent kinase (cdk) inhibitors	kinase inhibitor	breast cancer	2017	Novartis
Ripretinib	Other protein kinase inhibitors	kinase inhibitor	gastrointestinal stromal tumor	2020	Deciphera Pharmaceuticals
Rucaparib	Poly (adp-ribose) polymerase (parp) inhibitors	PARP-I	ovarian and prostate cancer	2016	Clovis Oncology, Inc.
Ruxolitinib	Janus-associated kinase (jak) inhibitors	kinase inhibitor	polycythemia vera, myelofibrosis and acute graft-versus-host disease	2011	Incyte Corporation
Selpercatinib	Kinase inhibitor	kinase inhibitor	non-small cell lung cancer, medullary thyroid cancer	2020	Eli Lilly
Selumetinib	kinase inhibitor (MEK1/2)	kinase inhibitor	Neurofibromatosis type 1	2020	Astra Zeneca
Sonidegib	Hedgehog pathway inhibitors	HP-I	basal cell carcinoma	2015	Novartis
Sorafenib	Other protein kinase inhibitors	kinase inhibitor	renal cell carcinoma	2005	Bayer
Sotorasib	KRAS inhibitor	kinase inhibitor	non-small cell lung cancer	2021	Amgen
Sunitinib	Other protein kinase inhibitors	kinase inhibitor	gastrointestinal stromal tumor and renal cell carcinoma	2006	Pfizer
Talazoparib	Poly (adp-ribose) polymerase (parp) inhibitors	PARP-I	breast cancer	2018	Pfizer
Tepotinib	Other protein kinase inhibitors	kinase inhibitor	non-small cell lung cancer	2021	EMD Serono
Tivozanib	Vascular endothelial growth factor receptor (vegfr) tyrosine kinase inhibitors	kinase inhibitor	renal cell carcinoma	2021	AVEO Pharmaceuticals
Tofacitinib	Selective immunosuppressants	kinase inhibitor	rheumatoid arthritis	2012	Pfizer
Trametinib	Kinase inhibitor	kinase inhibitor	metastatic melanoma	2013	GSK
Tucatinib	Kinase inhibitor	kinase inhibitor	breast cancer	2020	Seattle Genetics
Umbralisib	Kinase inhibitor	kinase inhibitor	follicular lymphoma and Marginal Zone Lymphoma	2021	TG Therapeutics
Upadacitinib	Selective immunosuppressants	kinase inhibitor	rheumatoid arthritis	2019	AbbVie
Vandetanib	Other protein kinase inhibitors	kinase inhibitor	medullary thyroid cancer	2011	Astra Zeneca
Vemurafenib	B-raf serine-threonine kinase (braf) inhibitors	kinase inhibitor	melanoma	2011	Genentech - Daïchi Sankyo
Vismodegib	Hedgehog pathway inhibitors	HP-I	basal cell carcinoma	2012	Genentech
Zanubrutinib	Bruton's tyrosine kinase inhibitor	kinase inhibitor	mantle cell lymphoma	2019	BeiGene
