# Supplementary material for: Involvement of Transporters in Intestinal Drug–Drug Interactions of Oral Targeted Anticancer Drugs Assessed by Changes in Drug Absorption Time
Source: Pharmaceutics. 2022 Nov 17;14(11):2493. doi: 10.3390/pharmaceutics14112493 (PMC9696196; doi:10.3390/pharmaceutics14112493)
Supplement: Supplementary file 1 [file pharmaceutics-14-02493-s001.zip › Table S2.pdf]

Table S2. Estimated concentration in the intestinal lumen at neutral pH (Igut, mM) for a series of oral targeted anticancer drugs.

Drug	MW (g/mol)	USP classification at neutral pH	Maximal solubility at neutral pH (mg/ml)	Usual dose per administration (mg)	Theoretical maximal concentration (dose / 250 ml, mg/ml)	ratio between maximal solubility and theroretical concentration	Igut, estimated concentration in the intestinal lumen at neutral pH (mM)
Abemaciclib	507	PI	0.1	150	0.6	6.00	0.20
Acalabrutinib	466	PI	0.1	100	0.4	4.00	0.21
Afatinib	486	S	100	40	0.16	0.00	0.33
Alectinib	483	PI	0.1	600	2.4	24.00	0.21
Alpelisib	442	PI	0.1	300	1.2	12.00	0.23
Avapritinib	499	PI	0.1	300	1.2	12.00	0.20
Axitinib	387	PI	0.1	5	0.02	0.20	0.05
Baricitinib	371	PI	0.1	2	0.008	0.08	0.02
Binimetinib	441	PI	0.1	45	0.18	1.80	0.23
Bosutinib	530	PI	0.1	500	2	20.00	0.19
Brigatinib	441	VS	1000	90	0.36	0.00	0.82
Cabozantinib	502	PI	0.1	140	0.56	5.60	0.20
Capmatinib	412	nd	nd	400	1.6	nd	nd
Ceritinib	558	PI	0.1	750	3	30.00	0.18
Cobimetinib	532	VSS	1	60	0.24	0.24	0.45
Crizotinib	450	PI	0.1	250	1	10.00	0.22
Dabrafenib	520	PI	0.1	150	0.6	6.00	0.19
Dacomitinib	470	PI	0.1	45	0.18	1.80	0.21
Dasatinib	489	PI	0.1	70	0.28	2.80	0.20
Duvelisib	417	PI	0.1	25	0.1	1.00	0.24
Enasidenib	473	PI	0.1	100	0.4	4.00	0.21
Encorafenib	540	PI	0.1	450	1.8	18.00	0.19
Entrectinib	561	PI	0.1	600	2.4	24.00	0.18
Erdafitinib	447	SS	10	8	0.032	0.00	0.07
Erlotinib	393	VSS	1	150	0.6	0.60	1.53
Fedratinib	525	PI	0.1	400	1.6	16.00	0.19
Fostamatinib	581	SS	10	100	0.4	0.04	0.69
Gefitinib	447	PI	0.1	250	1	10.00	0.22
Gilteritinib	553	SPS	33	120	0.48	0.01	0.87
Glasdegib	374	SS	10	200	0.8	0.08	2.14
Ibrutinib	441	PI	0.1	500	2	20.00	0.23
Idelalisib	415	PI	0.1	150	0.6	6.00	0.24
Imatinib	494	VS	1000	200	0.8	0.00	1.62
Infigratinib	560	PI	0.1	125	0.5	5.00	0.18
Ivosidenib	583	PI	0.1	500	2	20.00	0.17
Lapatinib	581	PI	0.1	1250	5	50.00	0.17
Larotrectinib	428	FS	1000	100	0.4	0.00	0.93
Lenvatinib	427	VSS	1	24	0.096	0.10	0.22
Lorlatinib	406	VSS	1	100	0.4	0.40	0.98
Midostaurin	571	PI	0.1	50	0.2	2.00	0.18
Neratinib	557	PI	0.1	240	0.96	9.60	0.18
Nilotinib	530	PI	0.1	400	1.6	16.00	0.19
Niraparib	320	SS	10	300	1.2	0.12	3.75
Olaparib	435	VSS	1	300	1.2	1.20	2.30
Osimertinib	500	VSS	1	80	0.32	0.32	0.64
Palbociclib	448	VSS	1	125	0.5	0.50	1.12
Pazopanib	438	PI	0.1	800	3.2	32.00	0.23
Pemigatinib	488	PI	0.1	13.5	0.054	0.54	0.11
Pexidartinib	418	PI	0.1	400	1.6	16.00	0.24
Ponatinib	533	PI	0.1	45	0.18	1.80	0.19
Pralsetinib	534	PI	0.1	400	1.6	16.00	0.19
Regorafenib	483	PI	0.1	160	0.64	6.40	0.21
Ribociclib	435	VSS	1	600	2.4	2.40	2.30
Ripretinib	510	PI	0.1	150	0.6	6.00	0.20
Rucaparib	323	SS	10	600	2.4	0.24	7.42
Ruxolitinib	306	VSS	1	20	0.08	0.08	0.26
Selpercatinib	526	VSS	1	160	0.64	0.64	1.22
Selumetinib	458	SS	10	25	0.1	0.01	0.22
Sonidegib	486	PI	0.1	200	0.8	8.00	0.21
Sorafenib	465	PI	0.1	400	1.6	16.00	0.22
sotorasib	561	PI	0.1	960	3.84	38.40	0.18
Sunitinib	399	S	100	50	0.2	0.00	0.50
Talazoparib	380	PI	10	1	0.004	0.00	0.01
Tepotinib	493	nd	nd	450	1.8	nd	nd
Tivozanib	455	PI	0.1	1.34	0.005	0.05	0.01
Tofacitinib	312	VSS	1	5	0.02	0.02	0.06
Trametinib	615	PI	0.1	2	0.008	0.08	0.01
Tucatinib	481	VSS	1	300	1.2	1.20	2.08
Umbralisib	572	PI	0.1	800	3.2	32.00	0.17
Upadacitinib	380	VSS	1	15	0.06	0.06	0.16
Vandetanib	475	PI	0.1	300	1.2	12.00	0.21
Vemurafenib	490	PI	0.1	960	3.84	38.40	0.20
Vismodegib	421	PI	0.1	150	0.6	6.00	0.24
Zanubrutinib	472	PI	0.1	160	0.64	6.40	0.21
