# Supplementary material for: Involvement of Transporters in Intestinal Drug–Drug Interactions of Oral Targeted Anticancer Drugs Assessed by Changes in Drug Absorption Time
Source: Pharmaceutics. 2022 Nov 17;14(11):2493. doi: 10.3390/pharmaceutics14112493 (PMC9696196; doi:10.3390/pharmaceutics14112493)
Supplement: Supplementary file 1 [file pharmaceutics-14-02493-s001.zip › Figure S1.pdf]

```python
#Display of program name and author
print("Ka solvation in a one-compartment oral model")
print("by @David MALNOË")

#Import of the module to calculate logarithms (math.log), exponentials (math.exp) and powers (math.pow)
import math

#Initiation of the variable allowing to return to the beginning of the program
continue = True

#As long as the 'continue' variable is considered true, the program returns to this point after calculating the ka and MAT
12 while continue:
while continue:
    print("________________________________________________________________")
    print("\nChoice of pharmacokinetic model:")
    print("     1. Monocompartmental model, single oral administration: analytical approach")
    print("     2. Monocompartmental model, single oral administration: iterative approach")
    print("     3. One-compartment model, repeated oral administration: iterative approach")
    print("     4. Leave \n")

#Choice of the appropriate pharmacokinetic model
    choice = input("Choice ? ")
    print("________________________________________________________________\n")

#Ka solvation for a monocompartmental model, with single oral administration according an analytical approach
    if choice == "1" :

    #User input of known variables: as long as the variables are not a floating point number, the program stops and displays 'Enter a number'
        test_var = 0
        while test_var == 0 :
            print("Please fill in the followinf parameters using . as decimal separator:")
            half_life= input("t1/2 (h) = ")
            tmax = input("tmax (h) = ")
            try:
                float(half_life)
                float(tmax)
                test_var = 1
                break
            except ValueError:
                print("\nEnter a nombre\n")

 # Conversion of variables into floating point number (float), definition and calculation of the intermediate variables necessary afterwards,
 initiation of Lambert's W function
```

```python
        ka = float(1)
        half_life= float(half_life)
        tmax = float (tmax)
        t = float(0)
        n = int(0)
        ke = math.log(2)/half_life
        X = ke * tmax
        Z = (-X)/(math.exp(X))
        W = float(1)

    #Solving ka via Lambert's W function in its main branch
        if X >= 1 :

        #Loop for estimating W according to the Halley method for 100 iterations
            while n < 100 :
                n=n+1
                W = W-((W*math.exp(W)-Z)/((math.exp(W)*(W+1))-(((W+2)*(W*math.exp(W)-Z))/(2*W+2))))

        #Calculation of ka and MAT, display of results
            ka = W/(-tmax)
            mat = 1/ka
            print("\n_______________________________________________________\n")
            print("Estimation of ka using the main branch W0 of Lambert's function by Halley's method:\n")
            print("ka = ",ka," h^-1")
            print("mat = ",mat," h")

    # Solving ka via Lambert's W function in its alternative branch
        else :

        #Estimation of W by algorithmic approach (a, b, c and d are intermediate variables making it easier to express W)
            alpha = float(0.3205)
            a = math.log(-Z)
            b = math.pow((-((1+a)/2)),0.5)
            c = math.pow((1+alpha*b),(-1))
            d = math.pow(alpha,(-1))
            W = a - (2*d*(1-c))

        #Calculation of ka and MAT, display of results
            ka = W/(-tmax)
            mat = 1/ka
            print("_______________________________________________________\n")
            print("Estimation of ka using the alternative branch W-1 of Lambert's function by logarithmic approach:\n")
            print("ka = ",ka," h^-1")
```

```python
            print("mat = ",mat," h")

    #If user input is not ok, OK, o, O, yes, y, YES, Y, then continue becomes false and exits loop, else returns to beginning of loop
        print("\n_________________________________________________________________\n")
        suite = input("Do you want to continue (yes/no) ?")
        if suite not in ("ok","OK","o", "O","yes","y","YES","Y"):
            continue = False
            fin = input("Thank you: press enter to exit")

#Resolution of ka for a monocompartmental model, with single oral administration according to an iterative approach
    elif choice == "2" :

    # User input of known variables: as long as the variables are not a floating point number, the program stops and displays 'Enter a number'
        test_var = 0
        while test_var == 0 :
            print("Please fill in the followinf parameters using . as decimal separator:")
            half_life= input("t1/2 (h) = ")
            tmax = input("tmax (h) = ")
            ka = input("Estimate ka (if unknown, enter 1) :")
            try:
                float(half_life)
                float(tmax)
                float(ka)
                test_var = 1
                break
            except ValueError:
                print("\nEnter a nombre\n")

    # Conversion of variables into floating point number (float), definition and initiation of t (estimate of tmax) and n (counter of the number of iterations), calculation of ke
        half_life= float(half_life)
        tmax = float(tmax)
        ka = float(ka)
        t = float(0)
        n = int(0)
        ke = math.log(2)/half_life

    # Loop calculating t until t is close to tmax true
        while t < (0.9999999 * tmax) or t > (1.00000001 * tmax) :
            n=n+1

```

```python
        # If t > tmax then ka is too small, and a new value of ka is assigned
            if t > tmax:
                ka = ka+(ka/8)

        # If t < tmax then ka is too large, and a new value of ka is assigned
            else:
                ka = ka-(ka/8)

        # Calculation of t with the new value of ka
            t = (math.log(ka/ke))/(ka-ke)

    #Calculation of MAT from the approximate value of ka and display of the results
        mat = 1/ka
        print("\n_______________________________________________________\n")
        print("Estimation of ka by iteration:\n")
        print("iteration = ",n)
        print("ka = ",ka," h^-1")
        print("mat = ",mat," h")

    #If user input is not ok, OK, o, O, yes, y, YES, Y, then continue becomes false and exits loop, else returns to beginning of loop
        print("\n________________________________________________________________\n")
        suite = input("Do you want to continue (yes/no) ? ")
        if suite not in ("ok","OK","o", "O","yes","y","YES","Y"):
            continue = False
            fin = input("Thank you: press enter to exit")

# Resolution of ka for a monocompartmental model, with repeated oral administration according to an iterative approach
    elif choice == "3" :

    # User input of known variables: as long as the variables are not a floating point number, the program stops and displays 'Enter a number'
        test_var = 0
        while test_var == 0 :
            print("Please fill in the followinf parameters using . as decimal separator décimal :")
            half_life= input("t1/2 (h) = ")
            tmax = input("tmax (h) = ")
            tau = input("tau (h) = ")
            ka = input("Estimate ka (if unknown, enter 1):")
            try:
                float(half_life)
                float(tmax)
                float(tau)
```

```
            float(ka)
            test_var = 1
            break
        except ValueError:
            print("\nEnter a nombre \n")

# Conversion of variables into floating point number (float), definition and initiation of t (estimate of tmax) and n (counter of the number of iteration), calculation of ke
    half_life= float(half_life)
    tmax = float(tmax)
    tau = float(tau)
    ka = float(ka)
    t = float(0)
    n = int(0)
    ke = math.log(2)/half_life

#Loop calculating t until t is close to tmax true
    while t < (0.9999999 * tmax) or t > (1.00000001 * tmax) :
        n=n+1

    #If t > tmax then ka is too small, and a new value of ka is assigned
        if t > tmax:
            ka = ka+(ka/8)

    # If t < tmax then ka is too large, and a new value of ka is assigned
        else:
            ka = ka-(ka/8)

    #Calculate t with the new valu of ka (a, b, A and B are intermediate variables to  facilitate the expression of t)
        a = -ke * tau
        b = -ka * tau
        A = ka * (1-math.exp(a))
        B = ke * (1-math.exp(b))
        t = (math.log(A/B))/(ka-ke)

#Calculation of MAT from the approximate value of ka and display of the results
    mat = 1/ka
    print("_______________________________________________________\n")
    print("Estimation of ka by iteration:\n")
    print("iteration = ",n)
    print("ka = ",ka," h^-1")
    print("mat = ",mat," h")
```

```python
    # If user input is not ok, OK, o, O, yes, y, YES, Y, then continue becomes false and exits loop, else returns to beginning of loop
        print("\n_________________________________________________________________\n")
        suite = input("Do you want to continue (yes/no) ? ")
        if suite not in ("ok","OK","o", "O","yes","y","YES","Y"):
            continue = False
            fin = input("Thank you: press enter to exit ")

    #Choice de quitter le programme
    elif choice == "4" :
        continue = False
        fin = input("Thank you: press enter to exit")

# Returns 'Error' if the model choice entry is not compliant (different from 1, 2, 3 or 4)
    else:
        print("Error : entry is not valid.")

    # If user input is not ok, OK, o, O, yes, y, YES, Y, then continue becomes false and exits loop, else returns to beginning of loop
        print("\n_________________________________________________________________\n")
        suite = input("Do you want to continue (yes/no) ? ")
        if suite not in ("ok","OK","o", "O","yes","y","YES","Y"):
            continue = False
            fin = input("Thank you: press enter to exit")

#End of the programm
print("\n_________________________________________________________________\n")
```
