# Supplementary material for: Involvement of Transporters in Intestinal Drug–Drug Interactions of Oral Targeted Anticancer Drugs Assessed by Changes in Drug Absorption Time
Source: Pharmaceutics. 2022 Nov 17;14(11):2493. doi: 10.3390/pharmaceutics14112493 (PMC9696196; doi:10.3390/pharmaceutics14112493)
Supplement: Supplementary file 1 [file pharmaceutics-14-02493-s001.zip › Figure S2.pdf]

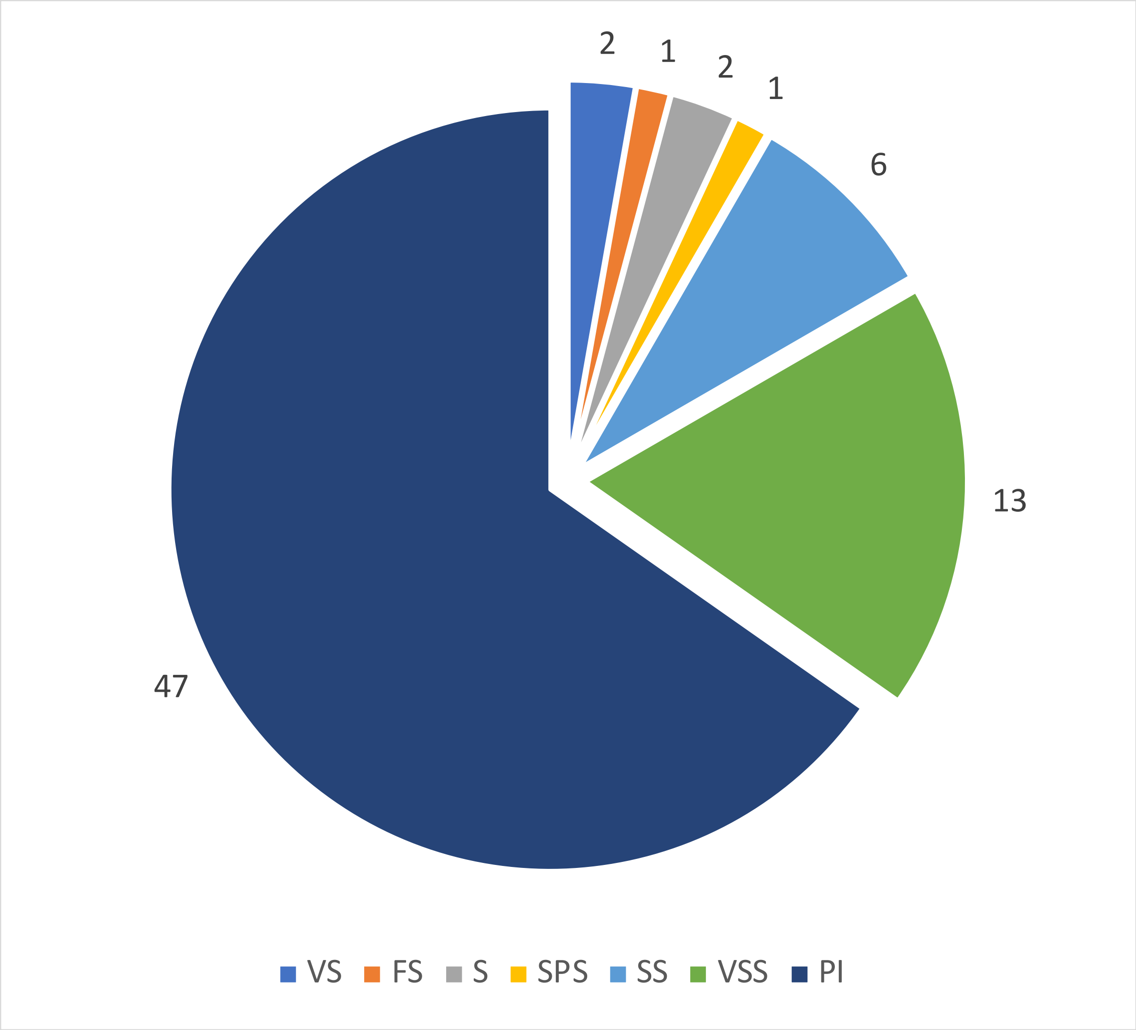

Figure S2. Distribution of the solubility at neutral pH classified according to the USP classification in a series of oral targeted anticancer drugs.
